# Prescribing trends and spontaneous reporting patterns of adverse drug reactions of ADHD medications in children and adolescents in germany: A retrospective analysis (2013–2022)

**DOI:** 10.1007/s00787-026-03031-5

**Published:** 2026-04-27

**Authors:** Diana Dubrall, Mona Reifferscheid, Karin Egberts, Stefanie Fekete, Antje Neubert, Hans W Rock, Paul Plener, Maike Below, Marcel Romanos, Matthias Schmid, Bernhardt Sachs

**Affiliations:** 1 Institute for Medical Biometry,Informatics and Epidemiology, University of Bonn, University Hospital Bonn, Bonn, Germany; 2 Research Division, Federal Institute for Drugs and Medical Devices (BfArM), Bonn, Germany; 3 Central Research Institute for Ambulatory Health Care in Germany, Berlin, Germany; 4 Department of Child and Adolescent Psychiatry, Psychosomatics andPsychotherapy, Center of Mental Health,, University Hospital of Wuerzburg, Wuerzburg, Germany; 5 Department of Child and adolescent Psychiatry, GGZ Reinier van Arkel, Hertogenbosch , The Netherlands; 6 Friedrich Alexander Universität, Erlangen Nürnberg, Medical Faculty, Department of Paediatric and Adolescents Medicine, Erlangen, Germany; 7 CIO Marburg GmbH, Lohra, Germany; 8 Department of Child and Adolescent Psychiatry, Medical University Vienna, Vienna, Austria; 9 Department of Child and Adolescent Psychiatry, University of Ulm, Ulm, Germany; 10 Department for Dermatology and Allergy, University Hospital RWTH Aachen, Aachen, Germany

**Keywords:** Adverse drug reactions, Attention deficit hyperactivity disorders, Children, Adolescent, Spontaneous reporting database, Drug prescription data

## Abstract

Attention-deficit hyperactivity disorder (ADHD) is a common psychiatric disorder in children and adolescents. Prescribing restrictions driven by concerns about overdiagnosis led to declining ADHD prescriptions between 2010 and 2016. The prevalence of adverse drug reactions (ADRs) remains unclear, as many studies did not account for dropouts due to ADRs. The aim of our study was to analyse the more recent ADHD prescriptions, along with the number of spontaneous ADR reports received for ADHD drugs. Outpatient ADHD drug prescriptions for patients ≤ 17 years between 01/01/2013 and 31/12/2022 were provided by the Central Research Institute of Ambulatory Health Care. Applying these inclusion criteria 1,119 spontaneous ADR reports of ADHD drugs were extracted from the European ADR database, EudraVigilance. Reporting rates were calculated as the number of ADHD reports per 100,000 outpatient ADHD drug prescriptions. After declining trends, the number of ADHD prescriptions increased again from 2020 onwards. In contrast, prescriptions for pre-school children increased throughout the study period. An increasing number of spontaneous ADHD reports was observed after 2017, with a slight decline between 2021 and 2022. Overall, reporting rates for methylphenidate (6.7) were clearly lower than for atomoxetine (17.8), lisdexamfetamine (22.2), and guanfacine (19.7); however initial differences converged over the course of the study. The increasing number of ADHD drug prescriptions in recent years appears to contrast with the recommended restrictions on ADHD drug use and the current treatment guidelines. Our study supports the recommendation to use methylphenidate as first-line treatment in children and adolescents.

**Supplementary Information:**

The online version contains supplementary material available at https://doi.org/10.1007/s00787-026-03031-5.

## Introduction

Inattention, hyperactivity and impulsivity are three of the core symptoms typically associated with the diagnosis of attention-deficit hyperactivity disorder (ADHD) [1]. These symptoms can vary regarding their severity. ADHD usually affects the social behaviour, individual performance and personal relationships of the patients. It is one of the most common diagnosed psychiatric disorders in children and adolescents in Germany, with a parent-reported lifetime diagnosis of 4.4%. Boys are diagnosed with ADHD around three times more often than girls [2].

In the case of moderate to severe symptoms of ADHD, pharmacotherapy is a recommended treatment option according to the German guidelines [1]. Drugs authorized for the treatment of ADHD in children and adolescents in Germany are psychostimulants (e.g., methylphenidate and amphetamines) and non-psychostimulant medications (e.g., atomoxetine and guanfacine). Methylphenidate is mostly used as first-line treatment, followed by amphetamines, atomoxetine and guanfacine which are considered as second-line options.

Due to an assumed over-diagnosis of ADHD and, thus, potential over-prescribing of ADHD drugs in the past, prescribing restrictions were released between 2009 and 2014 by a leading official health care body in Germany [3, 4]. Between 2009 and 2016 the number of children and adolescents with an ADHD diagnosis treated at least once with an ADHD drug declined from 50% (2010) to 44% (2016) [4]. In another study analysing ADHD drug prescriptions between 2012 and 2018 a slight decrease of ADHD drug prescriptions for children and adolescents could be observed, too [5]. In both studies this drop was especially apparent for methylphenidate, the most frequently prescribed ADHD drug in Germany, and for atomoxetine, but not for amphetamines [4, 5].

The prevalence of adverse drug reactions (ADRs) related to ADHD drugs is difficult to estimate as most of the studies analysing this topic did not consider dropouts from the respective study due to ADRs. Overall, most studies came to the result that ADRs of minor to moderate severity occur commonly in around 50–80% of the patients treated with ADHD drugs, while severe ADRs were either not observed or only in a few patients [6]. Regarding ADR reports referring to children and adolescents in general, one of our previous studies showed that methylphenidate and atomoxetine were the first and fourth most frequently reported suspected/interacting drugs between 01/2000 until 02/2019 [7], highlighting the importance to analyse these reports more deeply. In the past, dear-doctor letters and safety concerns were released for methylphenidate and atomoxetine [8–10]. These may have led to a stimulated reporting of ADRs associated with both drugs [11].

The first aim of our study was to analyse whether the more recent prescriptions of ADHD drugs increased again for children and adolescent in Germany. Secondly, we aimed to determine the number of spontaneously reported ADRs related to ADHD drugs referring to children and adolescents from Germany. Thirdly, we set the number of spontaneous ADR reports in relation to the number of drug prescriptions to analyse whether the reporting rates changed in the past for different strata (e.g., individual ADHD drugs).

## Methods

This is a non-interventional retrospective analysis of drug prescription data from the statutory health insurances from Germany and spontaneous ADR reports from the European ADR database EudraVigilance.

### Drug prescription data

Prescription data according to § 300 SGB V were provided by the Central Research Institute of Ambulatory Health Care [12] for patients ≤ 17 years with at least one outpatient prescription of a drug used for the treatment of ADHD (ATC N06BA (full list of drugs see Online Resource 1)) between 01/01/2013 and 31/12/2022. Thereby, both the annual number of ADHD drug prescriptions and the annual number of patients with ADHD drug prescriptions were requested. Notably, only outpatient ADHD drug prescriptions for patients with statutory health insurances (almost 90% of the German population) dispensed in German pharmacies are included. Inpatient prescriptions are not covered. In the number of ADHD drug prescriptions per year, one patient could be included multiple times e.g., in case of continuous or long-term ADHD drug therapy, whereas the number of patients with ADHD drug prescriptions represents the individuals exposed to ADHD drugs at least once during one calendar year.

### Eudravigilance

In Germany, ADRs can be reported spontaneously by health care professionals (HCP) such as physicians and pharmacists or non-health care professionals (non-HCP) like consumers and their relatives [13, 14]. These ADR reports are forwarded to and stored in EudraVigilance, the European ADR database of the European Medicines Agency (EMA) [15]. Besides the ADR reports from Germany, EudraVigilance also includes all spontaneous ADR reports received from the member states of the European Economic Area.

In accordance with the legal definition an ADR report is classified as serious, if the ADR was life-threatening, led to death, permanent disabilities, congenital anomalies, or hospitalization or prolongation thereof [13]. Notably, the legal definition may not correspond to the clinical definition of the severity of an ADR. Additionally, more than one seriousness criterion can be reported in one ADR report.

#### Identification of spontaneous reports

We identified all spontaneous ADR reports referring to children and adolescents (0–17 years) received between 01/01/2013 and 31/12/2022 from Germany in which centrally acting sympathomimetics of the ATC (Anatomical Therapeutic Chemical) class N06BA (full list of drugs see Online Resource 1) were reported as suspected/interacting (*n* = 1,244) (in the following: ADHD reports) [16].

We excluded all duplicates by case identifiers, reports describing intentional overdoses, misuses or suicide attempts, reports related to drug exposure during pregnancy, reports received from embryotox [17] and reports obtained from the KiDSafe study [18, 19]. Finally, our dataset consists of *n* = 1,119 ADHD reports. Notably, one patient could be included multiple times in case of several ADRs experienced by one patient reported at different dates.

The completeness of the structured information provided in the ADHD reports was measured by the vigiGrade completeness score originally established for the use in VigiBase [20]. Therefore, the score was slightly adapted to meet the structure of the ADR reports from EudraVigilance. According to the original algorithm, a threshold of ≥ 0.8 was defined for the designation of well-documented reports. The ADHD reports achieved a median vigiGrade completeness score of 0.48 [0.32–0.78]. Based on the median and interquartile range, the analysed ADHD reports fell below the threshold for well-documented reports.

**Fig. 1:**
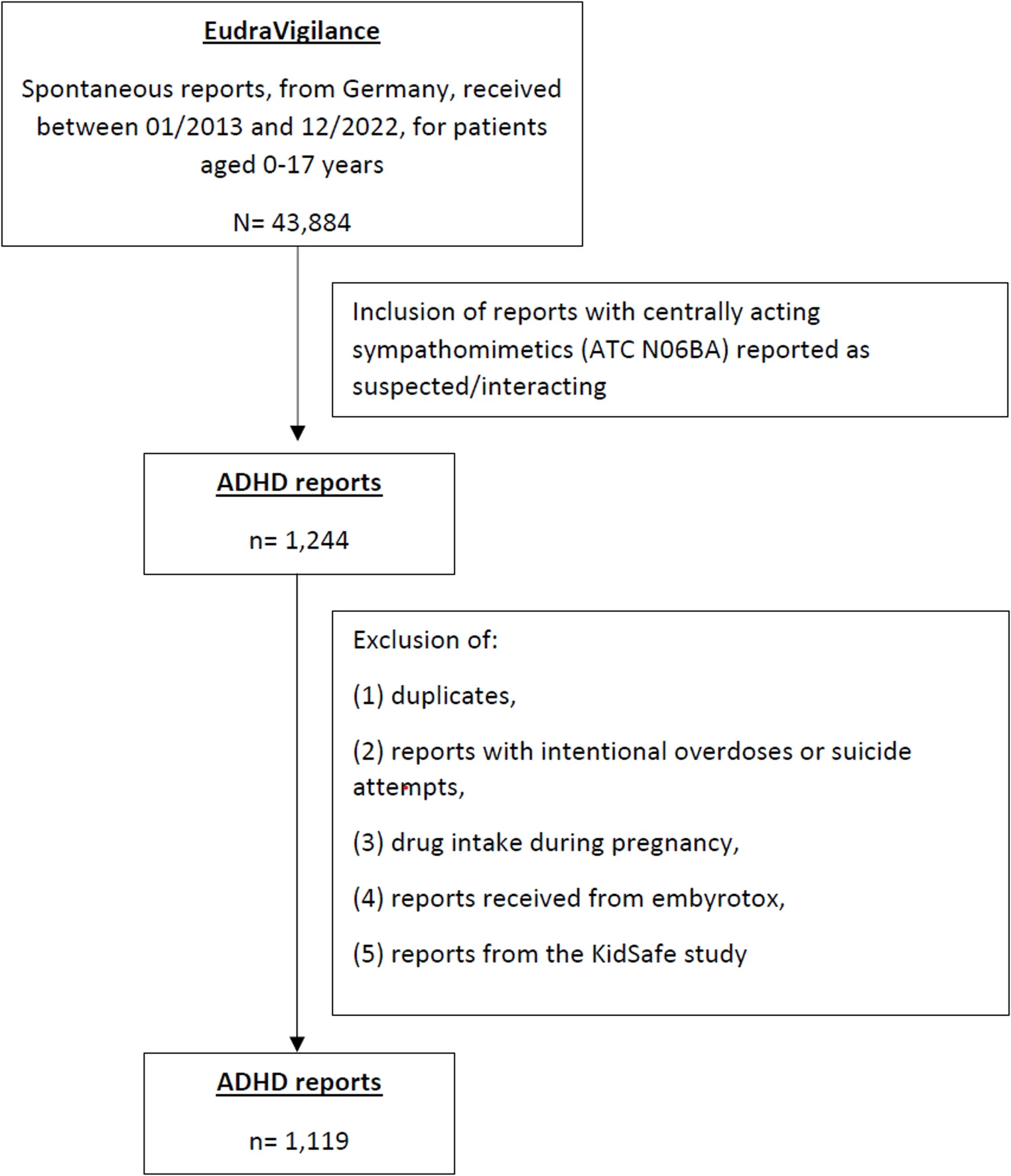
) Flow chart: identification of the ADHD reports

#### Analysis criteria

We determined the number of ADHD reports per year stratified by sex, age groups, reporter types and seriousness criteria.

Considering the sex of the patients, the stratifications females, males and unknown were used.

For the age groups, the patients were classified into pre-school children (< 6 years), school children (6–12 years), and adolescents (> 12 years).

Regarding the reporting sources, only the number of ADHD reports explicitly submitted by physicians, pharmacists, consumers, and other HCP were analysed. ADHD reports with more than one reporting source are included in the dataset but were not considered in the analysis of reporter types.

For the seriousness criteria, we considered whether the ADR report was classified as serious or non-serious and if hospitalisation or prolongation thereof was recorded.

#### Calculation of reporting rates

The number of spontaneous ADHD reports was divided by the number of outpatient ADHD drug prescriptions to calculate the so called “reporting rates”. In both, the numerator and denominator, patients could be included multiple times.

As a result, the reporting rates are presented as the number of ADHD reports per 100,000 prescriptions (1). For sensitivity analysis, we additionally calculated rates based on the number of spontaneous reports relative to the number of patients receiving ADHD drug prescriptions.

1 $$\:\begin{array}{c}\frac{Number\:of\:spontaneous\:ADHD\:reports}{Number\:of\:ADHD\:drug\:prescriptions}\ast\mathrm{100,000}\\=Number\:of\:ADHD\:reports\:per\:\mathrm{100,000}\:prescription\:per\:year\end{array}\\\\\\$$

#### Statistical analyses

For the statistical analyses R version 4.3.2 was used. The mean and median numbers of prescription counts, ADHD reports and reporting rates received between 2013 and 2022 with their standard deviations (SD) and interquartile ranges (IQR) were calculated for all stratifications.

Temporal trends of drug prescription counts were evaluated by negative binomial regression models. Annual changes are presented as percentages per year, along with rate ratios (RR) and corresponding 95% confidence intervals (CIs). Additionally, to identify possible breakpoints, Poisson regression models with joinpoint (segmented) regression (R package “segmented” [21]) were fitted to the data on drug prescriptions. For each interval before and after the breakpoint, annual percentage changes along with their RR and 95% CIs were estimated.

For temporal analysis of the reporting rates Poisson or negative binomial models with cubic splines were used to account for non-linear changes over time with drug prescription counts as offset to adjust for drug exposure (R package “splines” [22]). The 95% CIs of the spline effects were calculated to assess whether there are any differences across individual ADHD drugs or across sex- and age-strata. In case of overlapping CIs, no evidence for differences were found. These models were fitted for each individual ADHD drug separately, except for guanfacine. Drug prescription counts for guanfacine were available from 2020 onwards, thus, only three data points were available, which is why guanfacine was excluded from temporal trend analysis. Moreover, temporal trends for dexamfetamine could not be analysed in age- and sex-stratified analyses because no reports were available for some years.

## Results

### Drug prescription data

#### Number of drug prescriptions stratified by ADHD drugs

**Fig. 2:**
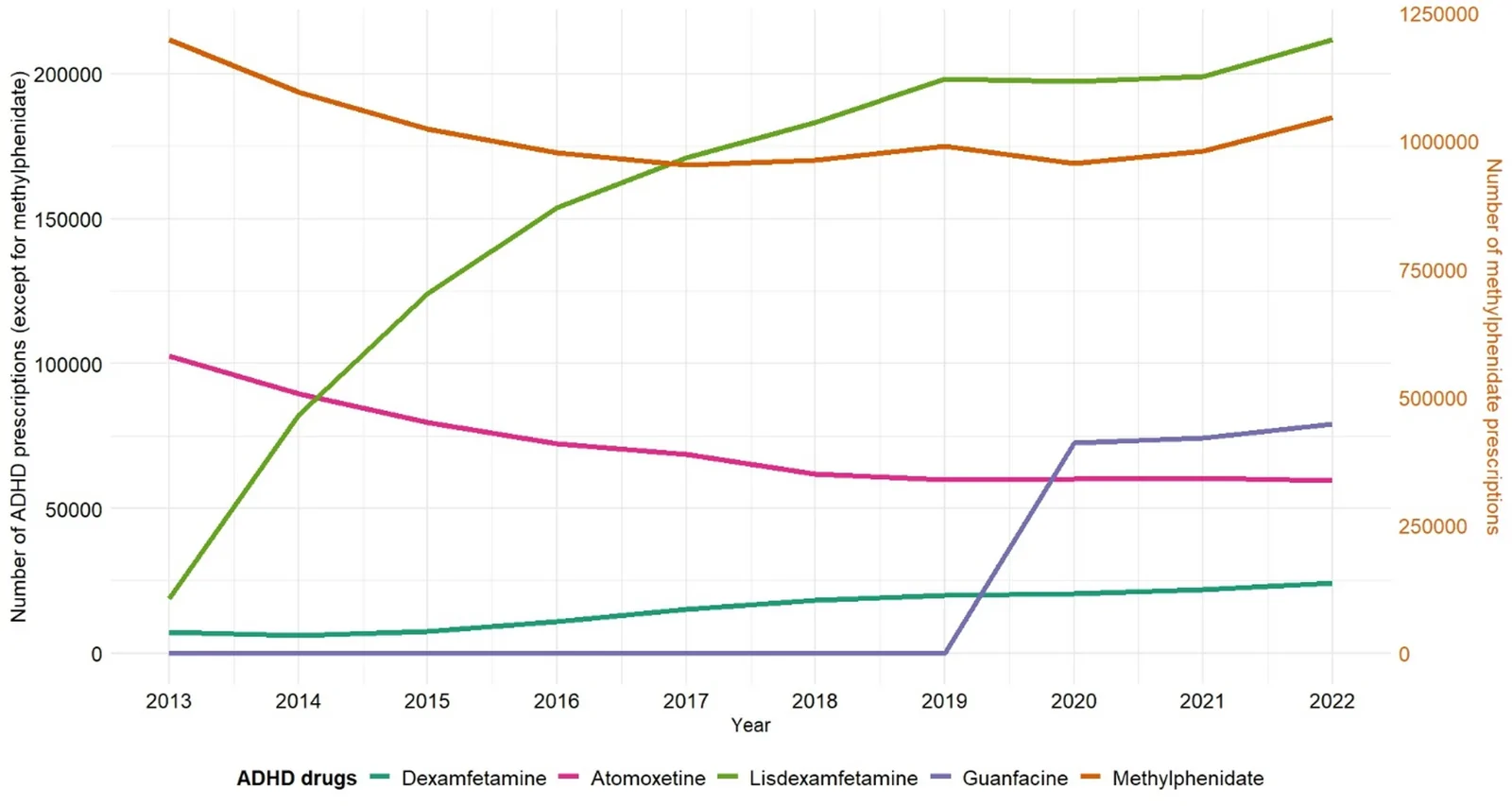
) Number of drug prescriptions stratified by ADHD drugs per year

Between 2013 and 2022 methylphenidate was the ADHD drug most frequently prescribed, followed by lisdexamfetamine, and atomoxetine (Table 1). Joinpoint regression analysis identified a breakpoint in 2016 for methylphenidate prescriptions. Before 2016, methylphenidate prescriptions decreased by 6.6% per year (RR = 0.934 [0.933–0.935]), whereas after 2016 they increased annually by 1.4% (RR = 1.014 [1.013–1.015]) (Fig. 2). Atomoxetine prescriptions declined by 5.7% per year (RR = 0.942 [0.928–0.957]), while lisdexamfetamine and dexamfetamine prescriptions increased by 17.1% (RR = 1.171 [1.076–1.276]) and 17.7% (RR = 1.177 [1.139–1.216]) per year. For guanfacine, data was available from 2020 onwards with slightly rising prescriptions through 2022. Between 2020 and 2022, guanfacine became the third most prescribed ADHD drug. The same tendencies as for the annual number of ADHD drug prescriptions were seen for the number of patients receiving ADHD drugs prescriptions (Online Resource 2 Table 1; Fig. 1).

**Table 1:** The cumulative number of ADHD drug prescriptions between 2013 and 2022

Rank	Number of ADHD drug prescriptions
1.	Methylphenidate (n= 10,185,760)
2.	Lisdexamfetamine (n= 1,540,274)
3.	Atomoxetine (n= 715,347)
4.	Guanfacine (n= 226,042)
5.	Dexamfetamine (n= 151,801)

Table 1 shows the total number of ADHD drug prescriptions between 2013 and 2022. Notably, Guanfacine received approval in 2015, probably explaining its lower counts. Data regarding guanfacine were available from 2020 onwards.

Figure 2 shows the number of prescriptions of ADHD drugs per year. Notably, the number of methylphenidate prescriptions is shown on the secondary y-axis. Guanfacine received approval in 2015, probably explaining its lower counts. Data regarding guanfacine were available from 2020 onwards.

#### Number of drug prescriptions stratified by age groups

Methylphenidate was the ADHD drug most frequently prescribed in pre-school children, school children, and adolescents (Online Resource 3 Table 2). The second most prescribed ADHD drugs were atomoxetine in pre-school children, and lisdexamfetamine in school children and adolescents.

The declining trends for methylphenidate prescriptions were seen in school children until 2016 (8.4% per year, RR = 0.916 [0.914–0.917]) and for adolescents until 2021 (4.8% per year, RR = 0.952 [0.952–0.953]), but its prescription increased for both afterwards, respectively (school children: 2.7% per year, RR = 1.027 [1.027–1.028]; adolescent: 10.6% per year, RR = 1.106 [1.101–1.111]) (Online Resource 3 Figs. 2 and 3). Compared to that, atomoxetine prescriptions decreased over the entire period in school children (4.9% per year, RR = 0.951 [0.935–0.967]) and adolescents (6.9% per year, RR = 0.931 [0.917–0.944]). In contrast, an increase of methylphenidate (4.7% per year, RR = 1.047 [1.035–1.060]) and atomoxetine (8.4% per year, RR = 1.084 [1.053–1.115]) prescriptions was observed for pre-school children (< 6 years) over the whole analysis period (Online Resource 3 Fig. 1). This was also apparent for the number of pre-school children with ADHD drug prescriptions (Online Resource 2 Fig. 2). Lisdexamfetamine prescriptions rose between 2019 and 2022 for pre-school children (17.6% per year, RR = 1.176 [1.113–1.243]), too, leading to even higher numbers in 2021 and 2022 compared to atomoxetine. Moreover, guanfacine was prescribed in pre-school children about as often as atomoxetine in 2021 and 2022, while its prescriptions were even higher for school children and adolescents as for atomoxetine from 2020 onwards.

#### Number of drug prescriptions stratified by sex

The ranking of the ADHD drugs prescribed was the same for females and males (Online Resource 4 Table 2). During the time course, methylphenidate prescriptions decreased for males until 2016 (6.9% per year, RR = 0.930 [0.930–0.931]), and slightly increased afterwards (0.8% per year, RR = 1.008 [1.008–1.009]) (Online Resource 4 Fig. 1). However, the total number in 2022 remained below the level observed in 2013. Compared to that, the number of methylphenidate prescriptions for females was almost the same again in 2022 as in 2013 (Online Resource 4 Fig. 2). For both sexes, atomoxetine prescriptions declined (females: 3.7% per year, RR = 0.963 [0.947–0.980]; males: 6.3% per year, RR = 0.937 [0.924–0.951]). The largest growth of prescriptions was noted for lisdexamfetamine for both sexes (females: 19.4% per year, RR = 1.194 [1.100-1.295]; males: 16.7% per year, RR = 1.167 [1.071–1.273]).

### Spontaneous reports

#### Number of ADHD reports stratified by ADHD drugs per year

Overall, *n* = 1,119 spontaneous ADR reports related to ADHD drugs were submitted for children and adolescents following our in- and exclusion criteria (Fig. 1). The number of ADHD reports increased between 2013 and 2022, but to a lower extent (1.2-fold; 2013: 90 reports; 2022: 108 reports) than the overall number of spontaneous ADR reports referring to children and adolescents (2.0-fold (2013: 596 reports; 2022: 1,172 reports) (Online Resource 5 Fig. 1). For both, a higher rise was observed between 2017 and 2018 (ADHD reports: 1.8-fold; all ADR reports: 2.1-fold), followed by decreasing numbers between 2020 and 2022 (both: 0.7-fold).

Between 2013 and 2022, the highest number of ADHD reports was received for methylphenidate (*n* = 673, 60.1%), followed by lisdexamfetamine (*n* = 240, 21.4%), atomoxetine (*n* = 123, 11.0%), guanfacine (*n* = 96, 8.6%), and dexamfetamine (*n* = 24, *n* = 2.1%) (Online Resource 5 Table 2).

**Fig. 3:**
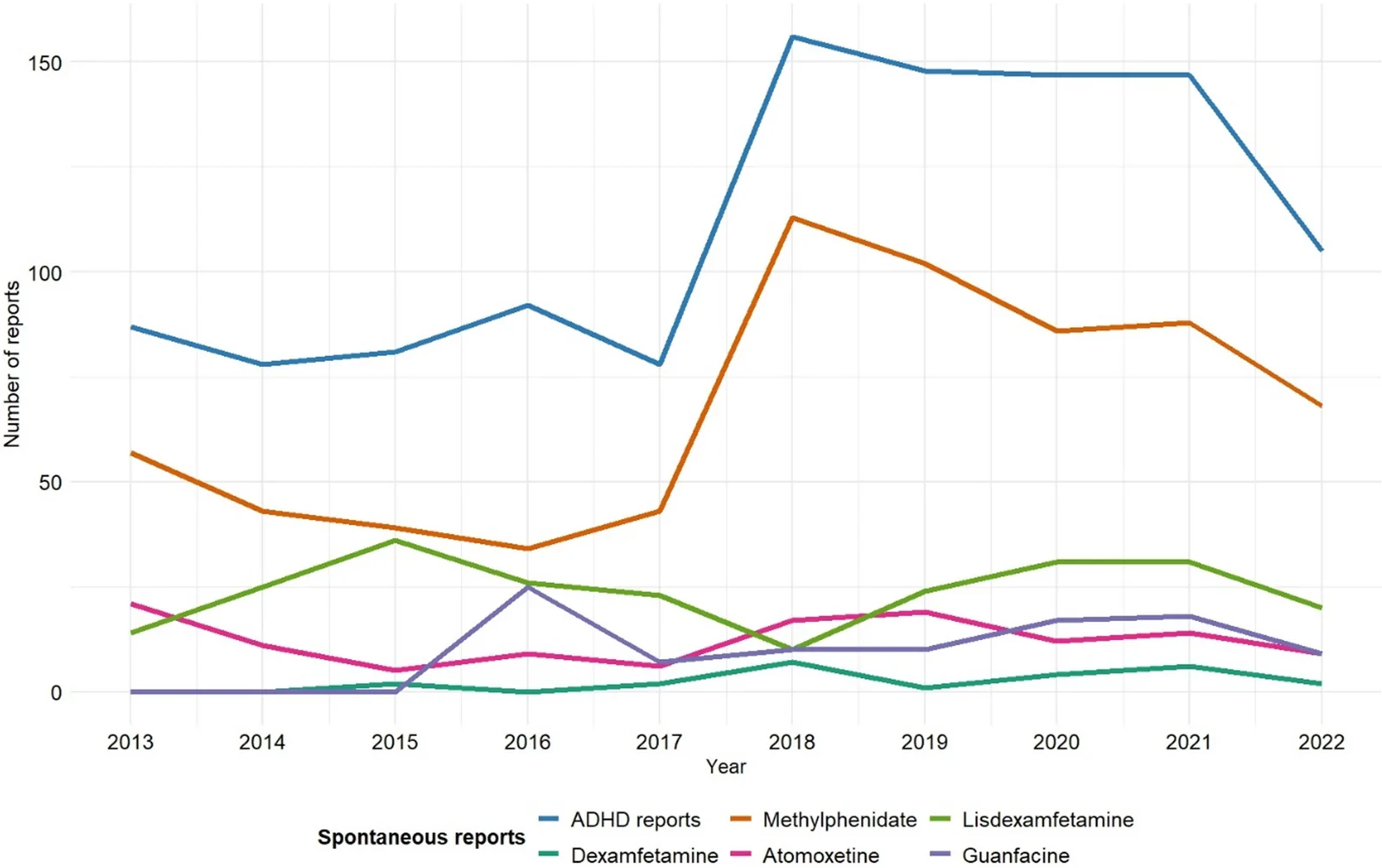
) Number of ADHD reports stratified by ADHD drugs per year

Stratified by the individual ADHD drugs reported as suspected/interacting, the increase between 2017 and 2018 was especially apparent for methylphenidate (2.6-fold), atomoxetine (2.8-fold), and dexamfetamine (3.5-fold) (Fig. 3). Additionally, the following decline between 2020 and 2022 was particularly observed for ADR reports related to methylphenidate than for the other ADHD drugs. Except for some small peaks, such as the higher number of reports reporting lisdexamfetamine as suspected/interacting in 2015 and guanfacine in 2016, the number of reports related to the other ADHD drugs besides methylphenidate were rather constant.

Figure 3 shows the number of spontaneous ADR reports for the individual ADHD drugs per year. Guanfacine received approval in 2015. The first ADR report related to guanfacine was submitted in 2016. Although dexamfetamine was approved in 2012, the first ADR report was forwarded in 2015.

#### Number of ADHD reports stratified by reporter type and seriousness per year

Almost half of the ADHD reports were reported by physicians (*n* = 544, 48.6%) (Online Resource 5 Table 2). 17.4% of the ADHD reports were received from consumers (*n* = 195), 7.5% from pharmacists (*n* = 84), and 4.2% from HCP (*n* = 47). 41.6% of the reports were classified as serious (*n* = 466) and 14.9% were coded with hospitalization or prolongation thereof.

Between 2017 and 2018, there was a pronounced increase in ADHD reports submitted by consumers (2.9-fold), and of those classified as non-serious (3.2-fold) (Online Resource 5 Fig. 2). Subsequently, the number of non-serious ADHD reports remained clearly higher (mean: 3.4 (± 1.0) times) than the number of ADHD reports classified as serious, which declined over time (0.3-fold) (Online Resource 5 Fig. 3). This decline was observed for all ADHD drugs (Online Resource 5 Fig. 4), but more prominently for reports from physicians and pharmacists, and to a lesser extent for those from consumers (Online Resource 5 Fig. 5). Despite the small numbers per ADR within the ADHD reports classified as serious, declining numbers were observed for some of the most frequently reported ADRs, including suicidal ideation, tachycardia, and heart rate increased. (Online Resource 5 Fig. 6). Along with the number of serious ADHD reports, the number of reports coded with hospitalisation or prolongation thereof (0.5-fold) declined, too.

#### Number of ADHD reports stratified by age groups and sex

About three quarters (76.0%, *n* = 850) of the ADHD reports referred to males (females: 20.3% (*n* = 227); unknown: 3.8% (*n* = 42)) (Online Resource 5 Table 2). Considering patient sex, the increase in reports for males between 2017 and 2018 contributed more to the overall rise than that observed for females (Online Resource 5 Fig. 7). Overall, the number of ADR reports referring to females was more constant over time. During the analysed period the number of ADR reports related to males was on average 4.0 (± 1.2) times higher than for females. In 2018, there was also a higher number of reports in which the sex of the patient was stated as unknown.

Regarding the age groups of patients, most reports referred to school children (63.2%, *n* = 707) and adolescents (36.4%, *n* = 407) (Online Resource 5 Table 2). Only 5 reports were related to pre-school children. The number of ADR reports referring to school children was, on average, 1.8 (± 0.5) times higher compared to those concerning adolescents (Online Resource 5 Fig. 8).

### Reporting rates

#### Reporting rates stratified by ADHD drugs

**Fig. 4:**
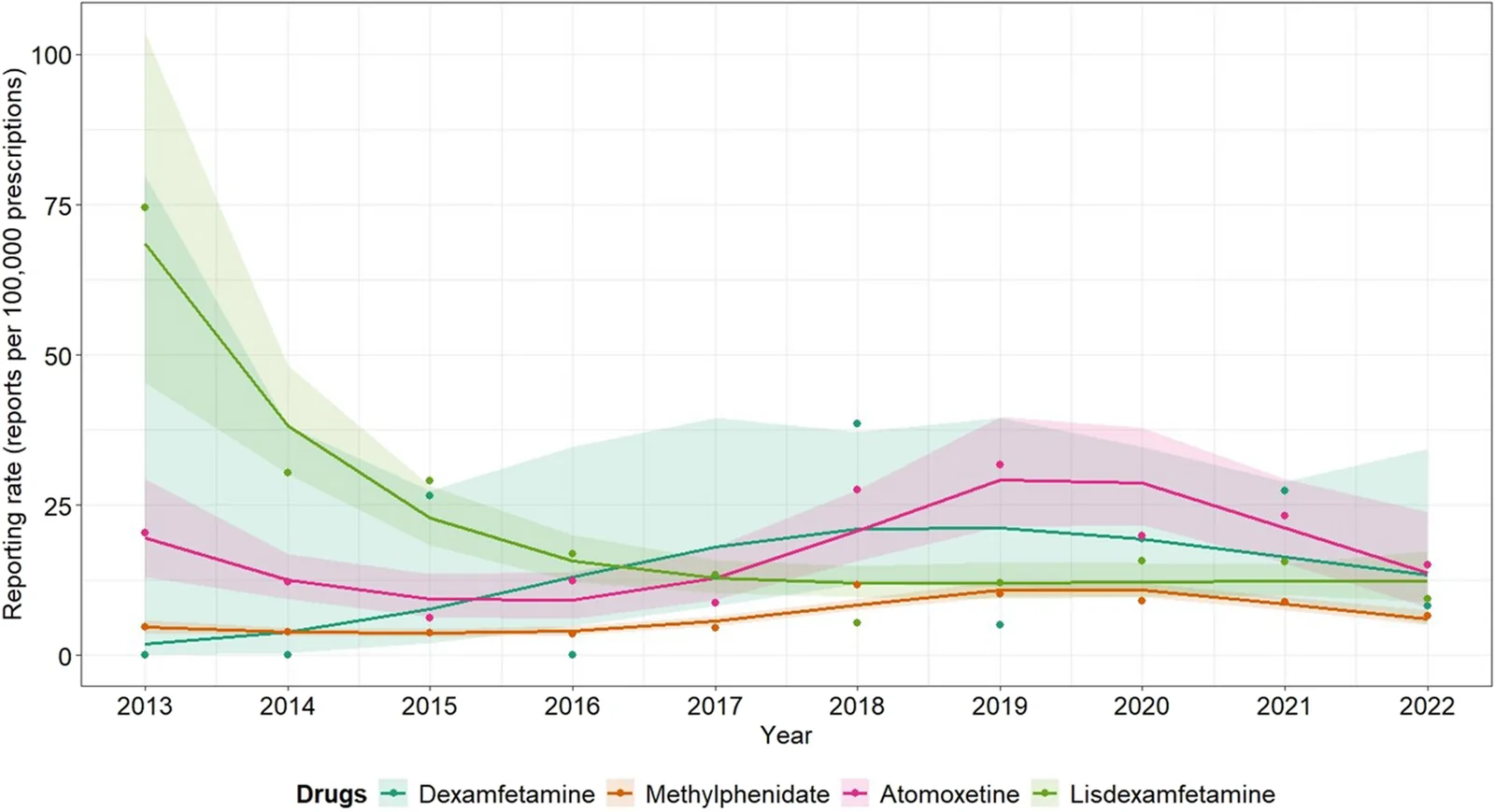
) Reporting rates per year stratified by individual ADHD drugs

Summarized for the years 2013 and 2022, the reporting rate was lowest for methylphenidate (mean: 6.7 (± 3.1)) (Online Resource 6 Table 2). The highest reporting rates were calculated for lisdexamfetamine (mean: 22.2 (± 19.9), and guanfacine (mean: 19.7 (± 7.2)) followed by atomoxetine (mean: 17.8 (± 8.2)), and dexamfetamine (mean: 17.3 (± 13.0)). Strikingly the reporting rates for lisdexamfetamine clearly decreased over time with higher rates between 2013 and 2015 than between 2016 and 2022 (Fig. 4). Overall, the reporting rate for methylphenidate was more consistent over the years. After 2017, the reporting rates for methylphenidate and lisdexamfetamine became more similar. Compared to methylphenidate, reporting rates for dexamfetamine and atomoxetine were rather inconsistent. This was particularly evident for dexamfetamine, given the wide CIs of the fitted splines. Between 2018 and 2021, reporting rates for atomoxetine and dexamfetamine were at a higher level than those of methylphenidate and lisdexamfetamine, with aligning tendencies in 2022. The calculated reporting rates in relation to the number of patients receiving ADHD drug prescription can be found in Online Resource 7.

Figure 4 shows the reporting rates for the individual ADHD drugs per year. For calculation of reporting rates, the number of spontaneous ADHD reports was divided by the number of outpatient ADHD prescriptions. For temporal analysis of the reporting rates Poisson or negative binomial models with cubic splines were used to account for non-linear changes over time with drug prescription counts as offset to adjust for drug exposure. These models were fitted for each individual ADHD drug separately, except for guanfacine. Drug prescription counts for guanfacine were available from 2020 onwards, thus, only three data points were available, which is why guanfacine was excluded from temporal trend analysis. The reporting rates of guanfacine can be found in Online Resource 6 Fig. 1. The reporting rates are given as the number of reports per 100,000 drug prescriptions.

#### Reporting rates stratified by ADHD drugs and seriousness

**Fig. 5:**
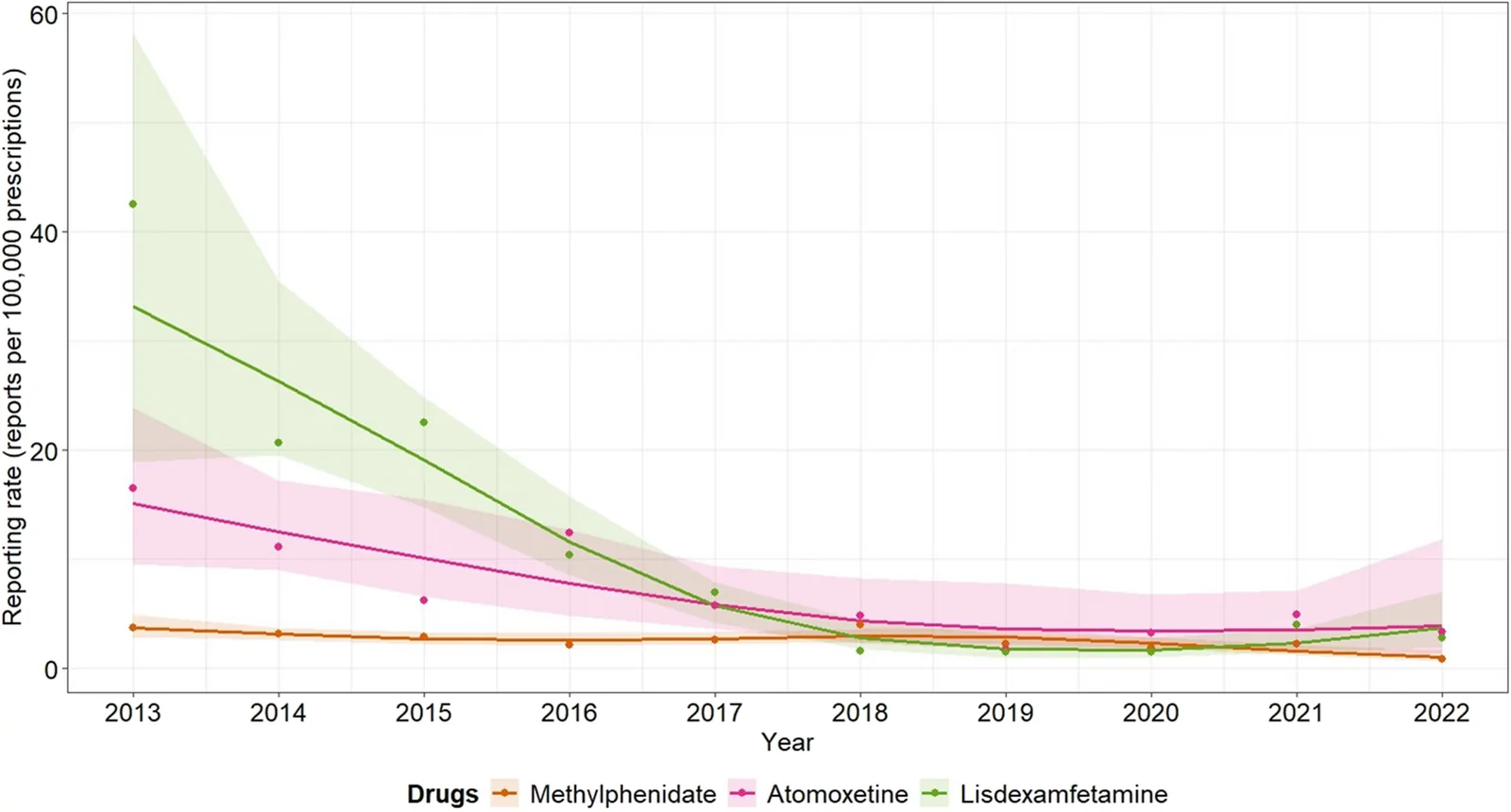
) Reporting rates of serious ADRs per year stratified by individual ADHD drugs

Methylphenidate also showed the lowest reporting rate for serious ADRs (mean: 2.6 (± 0.9)) (Online Resource 6 Table 2). Again, the highest reporting rate was calculated for lisdexamfetamine (mean: 11.5 (± 13.4), however, this was largely driven by higher rates between 2013 and 2016 (Fig. 5). Higher rates between 2013 and 2016 were also seen for atomoxetine. After 2018, the reporting rates for serious ADRs associated with methylphenidate, lisdexamfetamine, and atomoxetine converged. This tendency was confirmed by the overlapping CIs for the fitted splines of their reporting rates from 2018 onwards. In 2022, guanfacine showed a similar reporting rate than methylphenidate, atomoxetine, and lisdexamfetamine, although a higher reporting rate for serious ADRs was seen, especially for 2020 (Online Resource 6 Fig. 2).

Figure 5 shows the reporting rates of serious ADRs for the individual ADHD drugs per year. For calculation of reporting rates, the number of spontaneous ADHD reports was divided by the number of outpatient ADHD prescriptions. For temporal analysis of the reporting rates Poisson or negative binomial models with cubic splines were used to account for non-linear changes over time with drug prescription counts as offset to adjust for drug exposure. These models were fitted for each individual ADHD drug separately, except for guanfacine and dexamfetamine. Drug prescription counts for guanfacine were available from 2020 onwards, thus, only three data points were available, which is why guanfacine was excluded from temporal trend analysis. Serious ADR reports related to dexamfetamine were only reported in 2015 and 2020. Thus, only two data points were available, which is why dexamfetamine was excluded from temporal analysis. The reporting rates of guanfacine and dexamfetamine can be found in Online Resource 6 Fig. 2. The reporting rates are given as the number of reports per 100,000 drug prescriptions.

#### Reporting rates stratified by age groups and sex

Considering school children and adolescents separately, the reporting rates were higher for school children (mean values e.g., atomoxetine 19.6 (± 11.7) versus 15.3 (± 5.3); guanfacine 22.5 (± 8.2) versus 15.9 (± 14.3)), except for methylphenidate for which the reporting rates were quite similar (Online Resource 6 Table 3). It was striking that, especially between 2018 and 2019, the reporting rates for atomoxetine were clearly higher for school children than for adolescents, although the 95% CIs of the fitted splines overlapped (Online Resource 6 Figs. 3–6). Regarding the sexes, a higher reporting rate for females with a prescription of guanfacine was observed (mean: 24.0 (± 1.6) versus 18.2 (± 8.0)), while slightly higher reporting rates for males were noticed for lisdexamfetamine (mean: 21.7 (± 21.0) versus 18.2 (± 9.7)) (Online Resource 6 Table 4). For methylphenidate and atomoxetine, the overall reporting rates for females and males were similar (Online Resource 6 Figs. 7, 8, 9 and 10). The fitted splines and their 95% CIs indicated no apparent differences in reporting rates by age group or sex for methylphenidate, atomoxetine, or lisdexamfetamine, given the overlap of the CIs.

## Discussion

### Number of drug prescriptions

According to the German S3 guideline [1] methylphenidate is the first-line option for psychopharmacotherapy in ADHD, while amphetamines and non-stimulants are recommended only when initial treatment with methylphenidate proved unsuccessful or in case of relevant contraindications. Its first-line status explains the overall higher prescription numbers of methylphenidate compared to the other ADHD drugs in our study. The observed drop of methylphenidate and atomoxetine prescriptions between 2012 and 2017 might be associated with the concerns of over-diagnosis and over-treatment of ADHD resulting in recommendations to restrict their use [3, 4]. Another reason might be the updated treatment guideline [1] recommending pharmacological treatment only for moderate or severe ADHD and not as first-line therapy in children under 6 years. Likewise, the published warnings regarding serious ADRs could have influenced prescribing patterns (e.g., cardiovascular events – methylphenidate, suicidal ideations – atomoxetine [8–10]). Given these warnings and restrictions in use, switching from methylphenidate and atomoxetine to alternative ADHD drugs may have occurred to some extent and could have contributed to their drop in prescriptions [23]. Considering other studies performed in European countries, ADHD prescriptions in the Netherlands decreased [24] but increased in France [25], Denmark, Sweden and Norway [26] and almost doubled in England [27]. Based on sales data of ADHD drugs from 28 European countries [28], a continuous increase was seen between 2014 and 2022. However, the number of sales were lower as expected in the first year of the COVID-19 pandemic (2020) and higher as expected afterwards (2021–2022). In our study, the number of methylphenidate prescriptions already increased after 2018. Reasons for that could be a higher awareness of ADHD in the public (e.g., due to social media [29]), improvements in diagnosing ADHD, increasing mental health issues and worsening of ADHD symptoms in the time during and after the COVID-19-pandemic [28].

In contrast to methylphenidate, atomoxetine prescriptions declined constantly over the years in our analysis. One reason might be a less frequent prescribing due to lower effect sizes as seen in other studies [30]. Furthermore, the later onset of effects and the gaining of clinical experience with alternative ADHD drugs such as lisdexamfetamine may be further motives.

Despite the announced restrictions in Germany between 2009 and 2014 [3, 4], the number of prescriptions for lisdexamfetamine and dexamfetamine – although dexamfetamine prescription remained low – seemed to be unaffected. Lisdexamfetamine was approved in 2013 but became the second most prescribed ADHD drug from 2015 onwards exceeding the number of atomoxetine prescriptions– the second most prescribed ADHD drug until 2015 – which was already approved in 2005. This pattern was already seen in a previous analysis from Germany [4]. As in our study, lisdexamfetamine prescriptions increased in England [27], Norway and Sweden [26]. This was also the case in the Netherlands [24], although, in the Netherlands lisdexamfetamine plays a more subordinate role compared to the other European countries.

Guanfacine became the third most common prescribed drug in our study from 2020 onwards exceeding the number of atomoxetine prescriptions. According to the German guideline [1], guanfacine - alongside atomoxetine – is recommended as second-line treatment option if stimulants are ineffective or inappropriate for example due to comorbidities, an increased potential for abuse or other intolerances. Since guanfacine was approved in 2016, its prescription over time could not be covered in the previous study from Germany [4], but increasing prescriptions could also be observed in England [27] and Sweden [26].

In Germany, dexamphetamine seems to play a minor role in the treatment of ADHD in children and adolescents. According to literature dexamphetamine is more prone to abuse, and the higher likelihood of serious ADRs may possibly lead to a less frequent prescribing [31].

Although pharmacotherapy is not recommended as first-line treatment in pre-school children, it was striking that the number of ADHD prescriptions and the number of pre-school children exposed to ADHD drugs increased continuously between 2012 and 2022, especially for methylphenidate and atomoxetine, albeit the numbers remained at a low level compared to school children and adolescent. Diagnosing ADHD in children younger than 6 years is challenging and should only be carried out by specifically trained physicians [1]. This increase might also be attributed to greater familiarity among specialists with diagnosing and treating ADHD in pre-school children. Based on our data we cannot evaluate whether the prescriptions were carried out by specialists. Nevertheless, the increasing numbers over time despite the recommended restricted use appear contradictory. In the Netherlands there was a clearly negative trend in prescribing ADHD drugs to patients 0–6 years [24]. Further studies are needed to investigate this finding.

### Number of spontaneous reports

The observed growth of the number of spontaneous ADHD reports between 2017 and 2018 was probably attributed to the new pharmacovigilance legislation which came into force in 2012 with a translational phase until 2018 [32, 33]. In this legislation, changes in reporting obligations for pharmaceutical companies to forward non-serious ADR reports to EudraVigilance were established. The rising numbers of spontaneous ADR reports between 2017 and 2018 were already observed in other studies of our research group as well as discussed by the EMA in their annual reports [11, 33–35]. In our present study, as well as in these previous analyses, this increase was mostly caused by ADR reports from consumers and ADR reports classified as non-serious [33–35].

The decline of the number of spontaneous ADHD reports between 2021 and 2022 might be related to a preferential reporting for ADRs related to vaccines during the COVID-19 pandemic instead of drugs as assumed by others [36]. This decrease was also seen in other studies of our research group [37].

Considering the individual ADHD drugs separately, some peaks could be identified such as the higher number of methylphenidate and atomoxetine reports in 2013, lisdexamfetamine reports in 2015, and guanfacine reports in 2016. Regarding methylphenidate and atomoxetine, the published warnings [8–10] might have stimulated ADR reporting. This could explain the more frequent reporting of serious events such as suicidal ideation, tachycardia, and increased heart rate at the beginning of our analysis period as well as their subsequent decline thereafter. Likewise, the management of these ADRs could have improved, thereby reducing their occurrence or mitigating their course. In the case of lisadexamfetamine and guanfacine, which were approved in 2013 and 2016, respectively, the preferential reporting of ADRs associated with newly authorized drugs might be one reason [38]. The tendency for reports of ADRs to peak within the first few years after a drug is introduced to the market and then decline over time is called the “Weber effect” [39]. This pattern is often attributed to heightened awareness, stimulated reporting, and closer monitoring shortly after approval rather than a true change in the drug’s safety profile. However, the “Weber effect” remains of debate as it could not be replicated in other studies [40, 41].

### Reporting rates

Overall, the ranking of the ADHD drugs by ADR report counts reflects their prescribing frequency underscoring the importance of accounting for prescription data and calculating reporting rates when interpreting spontaneous ADR report analyses as previously described in literature [42]. In our study, the lowest reporting rate was calculated for methylphenidate, however, the reporting rates for methylphenidate and lisdexamfetamine became more similar over time. Thus, a reporting bias e.g., “Weber effect” in the beginning of our analysis period cannot be ruled out. Guanfacine and amphetamines were less well tolerated than placebo in a network meta-analysis investigating dropouts due to ADRs related to ADHD drugs [30]. However, the comparability to our results is limited as the entire group of amphetamines was considered but not the individual amphetamines. In our study, the reporting rate for guanfacine was clearly higher compared to methylphenidate, however, only three data point were available, limiting the reliability of this finding. Moreover, taking into account the Weber effect and the convergence observed in 2022, further research is required to examine this observation.

Likewise, as in England [27], methylphenidate reached the lowest reporting rate for serious ADRs compared to the other ADHD drugs in our study. In England, reporting rates for serious ADRs were also declining over time as in our study with converging tendencies towards methylphenidate, although the rates remained the highest for guanfacine.

Based on our results, it may be assumed that younger children (≤ 12 years) may be more prone to develop ADRs related to ADHD drugs compared to adolescents, although CIs of the fitted splines mostly overlapped. Others observed higher rates of ADRs especially in preschool children [43]. In our analysis, the small number of ADHD reports referring to pre-school children (*n* = 5) does not allow any reliable conclusions.

Regarding boys and girls, symptoms of ADHD and treatment effects of ADHD drugs might differ. Less is known about sex-differences regarding their safety profile [44]. In our study, only minor differences were observed regarding the reporting rates between females and males. Previous results of higher reporting rates for methylphenidate in females aged 13–17 years and for atomoxetine in males aged 7–12 years [7] could not be replicated in our study. Some studies have reported differences in the types of ADRs, an aspect that was beyond the scope of our analysis [45].

### Limitations

The available drug prescription data include patients with statutory health insurances, accounting for roughly 90% of the German population [12]. However, the number of prescriptions might not reflect the actual use e.g., in case of treatment non-compliance. Additionally, small numbers of total prescriptions in the dataset (≤ 30) could not be delivered for the requested analysis due to data protection requirements. This might be apparent for guanfacine which was approved in 2016 but only included from 2019 onwards in our requested data. ICD-10 diagnoses were not considered, thus, some of the ADHD drugs may not have been used for the treatment of ADHD. However, the number of patients using ADHD drugs for the treatment of narcolepsy is expected to be small. The length of therapy intervals as well as the number of prescribed defined daily doses (e.g., due to changes in package sizes) may affect the prescribing frequency and thus the number of prescriptions. The total number of drug prescriptions in each calendar year was unknown; thus, we could not adjust our regression models to population references like the number of insured individuals.

Regarding the spontaneous ADR reports, the unknown extent of underreporting [46] which might also differ between specific ADHD drugs [38] is one of the major limitations. On the other hand, published warnings [8–10] about serious ADRs may also have led to stimulated reporting. Moreover, reporting bias (e.g., “Weber effect”) could not be ruled out. All these factors may have introduced bias into the calculation of reporting rates and should be considered when interpreting the results of our analysis. Notably, all spontaneous ADRs reports represent suspected cases of ADRs. An individual case assessment regarding the causal relationship between the reported suspected drug and the ADR was not performed.

## Conclusion

The increasing number of ADHD drug prescriptions in recent years, particularly for pre-school children, appears to contrast with the recommended restrictions on ADHD drug use, especially in pre-school children, as outlined in current treatment guidelines. From a drug safety perspective, ADRs were reported less frequently for methylphenidate compared to the other ADHD drugs, supporting the recommendation to use methylphenidate as first-line treatment in children and adolescents.

## Supplementary Information

Below is the link to the electronic supplementary material.

Supplementary Material 1

Supplementary Material 2

Supplementary Material 3

Supplementary Material 4

Supplementary Material 5

Supplementary Material 6

Supplementary Material 7

## Data Availability

The pseudonymised ADR reports from EudraVigilance are not publicly accessible due to data protection requirements. Distinct levels of access are provided for various stakeholders (https://www.ema.europa.eu/en/human-regulatory/research-development/pharmacovigilance/eudravigilance/access-eudravigilance-data). Being one of the competent authorities in Germany, the highest level of access is granted to the Federal Institute for Drugs and Medical Devices (BfArM). Nevertheless, even with the lowest access level, researchers can perform the same analysis in EudraVigilance (EV) with aggregated data (public access: www.adrreports.eu/en/index.html). For further information regarding processing personal data in the context of the operation of EudraVigilance Human we refer to the European Medicines Agency’s Data Protection Notice for EudraVigilance Human.
