# Supplementary material for: Prescribing trends and spontaneous reporting patterns of adverse drug reactions of ADHD medications in children and adolescents in germany: A retrospective analysis (2013–2022)
Source: Eur Child Adolesc Psychiatry. 2026 Apr 27;35(8):2593–605. doi: 10.1007/s00787-026-03031-5 (PMC13652941; doi:10.1007/s00787-026-03031-5)
Supplement: Supplementary file 1 [file 787_2026_3031_MOESM1_ESM.docx]

**Title: Prescribing Trends and Spontaneous Reporting Patterns of Adverse Drug Reactions of ADHD Medications in Children and Adolescents in Germany: A Retrospective Analysis (2013-2022)**

**Journal: European Child & Adolescent Psychiatry**

**Authors:**

Diana Dubrall^1,2^, Mona Reifferscheid^3^, Karin Egberts^4,5^, Stefanie Fekete^4^, Antje Neubert^6^, Hans W Rock^7^, Paul Plener^8,9^, Maike Below^3^, Marcel Romanos^4^, Matthias Schmid^1^, Bernhardt Sachs^2,10^

^1^ University of Bonn, University Hospital Bonn, Institute for Medical Biometry, Informatics and Epidemiology, Bonn, Germany

^2^ Research Division, Federal Institute for Drugs and Medical Devices (BfArM), Bonn, Germany

^3^ Central Research Institute for Ambulatory Health Care in Germany, Berlin, Germany

^4^ Department of Child and Adolescent Psychiatry, Psychosomatics and Psychotherapy, Center of Mental Health, University Hospital of Wuerzburg, Wuerzburg, Germany

^5^ Department of Child and adolescent Psychiatry, GGZ Reinier van Arkel, ‘s-Hertogenbosch, The Netherlands

^6^ Friedrich Alexander Universität, Erlangen Nürnberg, Medical Faculty, Department of Paediatric and Adolescents Medicine, Erlangen, Germany

^7^ CIO Marburg GmbH, Lohra, Germany

^8^ Department of Child and Adolescent Psychiatry, Medical University Vienna, Vienna, Austria

^9^ Department of Child and Adolescent Psychiatry, University of Ulm, Ulm, Germany

^10^ Department for Dermatology and Allergy, University Hospital RWTH Aachen, Aachen, Germany

**Corresponding author:**

Dr. Diana Dubrall

University of Bonn, University Hospital Bonn, Institute for Medical Biometry, Informatics and Epidemiology, Bonn, Germany

Research Division, Federal Institute of Drugs and Medical Devices (BfArM), Bonn, Germany

E-mail: Diana.Dubrall@bfarm.de

**Online Resource 1) List of drugs (ATC N06BA).**

| N06BA01 | Amfetamine |
| --- | --- |
| N06BA02 | Dexamfetamine |
| N06BA03 | Metamfetamine |
| N06BA04 | Methylphenidate |
| N06BA05 | Pemoline |
| N06BA06 | Fencamfamin |
| N06BA08 | Fenozolone |
| N06BA09 | Atomoxetine |
| N06BA10 | Fenetylline |
| N06BA11 | Dexmethylphenidate |
| N06BA12 | Lisdexamfetamine |
| N06BA15 | Dexmethylphenidate and Sexdexmethylphenidate |
| N06BA16 | Amfetaminil |
| N06BA17 | Mesocarb |
| N06BA21 | Guanfacine |
