# Supplementary material for: Prescribing trends and spontaneous reporting patterns of adverse drug reactions of ADHD medications in children and adolescents in germany: A retrospective analysis (2013–2022)
Source: Eur Child Adolesc Psychiatry. 2026 Apr 27;35(8):2593–605. doi: 10.1007/s00787-026-03031-5 (PMC13652941; doi:10.1007/s00787-026-03031-5)
Supplement: Supplementary file 2 [file 787_2026_3031_MOESM2_ESM.docx]

**Title: Prescribing Trends and Spontaneous Reporting Patterns of Adverse Drug Reactions of ADHD Medications in Children and Adolescents in Germany: A Retrospective Analysis (2013-2022)**

**Journal: European Child & Adolescent Psychiatry**

**Authors:**

Diana Dubrall^1,2^, Mona Reifferscheid^3^, Karin Egberts^4,5^, Stefanie Fekete^4^, Antje Neubert^6^, Hans W Rock^7^, Paul Plener^8,9^, Maike Below^3^, Marcel Romanos^4^, Matthias Schmid^1^, Bernhardt Sachs^2,10^

^1^ University of Bonn, University Hospital Bonn, Institute for Medical Biometry, Informatics and Epidemiology, Bonn, Germany

^2^ Research Division, Federal Institute for Drugs and Medical Devices (BfArM), Bonn, Germany

^3^ Central Research Institute for Ambulatory Health Care in Germany, Berlin, Germany

^4^ Department of Child and Adolescent Psychiatry, Psychosomatics and Psychotherapy, Center of Mental Health, University Hospital of Wuerzburg, Wuerzburg, Germany

^5^ Department of Child and adolescent Psychiatry, GGZ Reinier van Arkel, ‘s-Hertogenbosch, The Netherlands

^6^ Friedrich Alexander Universität, Erlangen Nürnberg, Medical Faculty, Department of Paediatric and Adolescents Medicine, Erlangen, Germany

^7^ CIO Marburg GmbH, Lohra, Germany

^8^ Department of Child and Adolescent Psychiatry, Medical University Vienna, Vienna, Austria

^9^ Department of Child and Adolescent Psychiatry, University of Ulm, Ulm, Germany

^10^ Department for Dermatology and Allergy, University Hospital RWTH Aachen, Aachen, Germany

**Corresponding author:**

Dr. Diana Dubrall

University of Bonn, University Hospital Bonn, Institute for Medical Biometry, Informatics and Epidemiology, Bonn, Germany

Research Division, Federal Institute of Drugs and Medical Devices (BfArM), Bonn, Germany

E-mail: Diana.Dubrall@bfarm.de

**Online Resource 2) Analyses of patients receiving ADHD drug prescriptions.**

**Online Resource 2 Table 1) Cumulative number of patients with ADHD drug prescriptions** **between 2013 and 2022.**

| Rank | Number of patients with ADHD drug prescriptions |
| --- | --- |
| 1.  2.  3.  4.  5. | Methylphenidate (n= 1,797,417)  Lisdexamfetamine (n= 277,555)  Atomoxetine (n= 119,992)  Dexamfetamine (n= 34,102)  Guanfacine (n= 32,919) |

Online Resource 2 Table 1 shows the total number of patients with ADHD drug prescriptions between 2013 and 2022. Notably, Guanfacine received approval in 2015, which may explain its lower counts. Data regarding guanfacine were available from 2020 onwards.

**Online Resource 2 Figure 1) Number of patients with drug prescriptions stratified by individual ADHD drugs.**

Online Resource 2 Figure 1 shows the number of patients with prescriptions of ADHD drugs per year. Notably, the number of patients with methylphenidate prescriptions is shown on the secondary y-axis. Guanfacine received approval in 2015, which may explain its lower counts. Data regarding guanfacine were available from 2020 onwards.

**Online Resource 2 Table 2) Cumulative number of patients with ADHD drug prescriptions between 2013 and 2022 stratified by age groups.**

| Rank | Pre-school children (< 6 years) | School children (6-12 years) | Adolescents (13-17 years) |
| --- | --- | --- | --- |
| 1.  2.  3.  4.  5. | Methylphenidate (n= 5,499)  Lisdexamfetamine (n= 305)  Atomoxetine (n= 250)  Guanfacine (n= 132) | Methylphenidate (n= 1,002,847)  Lisdexamfetamine (n= 140,220)  Atomoxetine (n= 65,734)  Guanfacine (n= 20,020)  Dexamfetamine (n= 19,636) | Methylphenidate (n= 789,071)  Lisdexamfetamine (n= 137,030)  Atomoxetine (n= 54,008)  Dexamfetamine (n= 14,739)  Guanfacine (n= 12,767) |

Online Resource 2 Table 2 shows the total number of patients with ADHD drug prescriptions between 2013 and 2022 for the three age groups of pre-school children, school children and adolescents. Notably, Guanfacine received approval in 2015, which may explain its lower counts. Data regarding guanfacine were available from 2020 onwards.

**Online Resource 2 Figure 2) Number of pre-school children with drug prescriptions stratified by ADHD drug.**

Online Resource 2 Figure 2 shows the number of pre-school children with prescriptions of ADHD drugs per year. Notably, the number of patients with methylphenidate prescriptions is shown on the secondary y-axis. Guanfacine received approval in 2015, which may explain its lower counts. Data regarding guanfacine were available from 2020 onwards.

**Online Resource 2 Figure 3) Number of school children with drug prescriptions stratified by ADHD drugs.**

Online Resource 2 Figure 3 shows the number of school children with prescriptions of ADHD drugs per year. Notably, the number of patients with methylphenidate prescriptions is shown on the secondary y-axis. Guanfacine received approval in 2015, which may explain its lower counts. Data regarding guanfacine were available from 2020 onwards.

**Online Resource 2 Figure 4) Number of adolescents with drug prescriptions stratified by ADHD drugs.**

Online Resource 2 Figure 4 shows the number of adolescents with prescriptions of ADHD drugs per year. Notably, the number of patients with methylphenidate prescriptions is shown on the secondary y-axis. Guanfacine received approval in 2015, which may explain its lower counts. Data regarding guanfacine were available from 2020 onwards.

**Online Resource 2 Table 3) Cumulative number of females and males with drug prescriptions between 2013 and 2022.**

| Rank | Females | Males |
| --- | --- | --- |
| 1.  2.  3.  4.  5. | Methylphenidate (n= 393,486)  Lisdexamfetamine (n= 50,552)  Atomoxetine (n= 26,365)  Dexamfetamine (n= 6,922)  Guanfacine (n= 5,738) | Methylphenidate (n= 1,403,931)  Lisdexamfetamine (n= 227,003)  Atomoxetine (n= 93,627)  Guanfacine (n= 27,181)  Dexamfetamine (n= 27,180) |

Online Resource 2 Table 3 shows the total number of females and males with ADHD drug prescriptions between 2013 and 2022. Notably, Guanfacine received approval in 2015, which may explain its lower counts. Data regarding guanfacine were available from 2020 onwards.

**Online Resource 2 Figure 5) Number of males with ADHD drug prescriptions.**

Online Resource 2 Figure 5 shows the number of males with prescriptions of ADHD drugs per year. Notably, the number of patients with methylphenidate prescriptions is shown on the secondary y-axis. Guanfacine received approval in 2015, which may explain its lower counts. Data regarding guanfacine were available from 2020 onwards.

**Online Resource 2 Figure 6) Number of females with ADHD drug prescriptions.**

Online Resource 2 Figure 6 shows the number of females with prescriptions of ADHD drugs per year. Notably, the number of patients with methylphenidate prescriptions is shown on the secondary y-axis. Guanfacine received approval in 2015, which may explain its lower counts. Data regarding guanfacine were available from 2020 onwards.
