# Supplementary material for: Prescribing trends and spontaneous reporting patterns of adverse drug reactions of ADHD medications in children and adolescents in germany: A retrospective analysis (2013–2022)
Source: Eur Child Adolesc Psychiatry. 2026 Apr 27;35(8):2593–605. doi: 10.1007/s00787-026-03031-5 (PMC13652941; doi:10.1007/s00787-026-03031-5)
Supplement: Supplementary file 3 [file 787_2026_3031_MOESM3_ESM.docx]

**Title: Prescribing Trends and Spontaneous Reporting Patterns of Adverse Drug Reactions of ADHD Medications in Children and Adolescents in Germany: A Retrospective Analysis (2013-2022)**

**Journal: European Child & Adolescent Psychiatry**

**Authors:**

Diana Dubrall^1,2^, Mona Reifferscheid^3^, Karin Egberts^4,5^, Stefanie Fekete^4^, Antje Neubert^6^, Hans W Rock^7^, Paul Plener^8,9^, Maike Below^3^, Marcel Romanos^4^, Matthias Schmid^1^, Bernhardt Sachs^2,10^

^1^ University of Bonn, University Hospital Bonn, Institute for Medical Biometry, Informatics and Epidemiology, Bonn, Germany

^2^ Research Division, Federal Institute for Drugs and Medical Devices (BfArM), Bonn, Germany

^3^ Central Research Institute for Ambulatory Health Care in Germany, Berlin, Germany

^4^ Department of Child and Adolescent Psychiatry, Psychosomatics and Psychotherapy, Center of Mental Health, University Hospital of Wuerzburg, Wuerzburg, Germany

^5^ Department of Child and adolescent Psychiatry, GGZ Reinier van Arkel, ‘s-Hertogenbosch, The Netherlands

^6^ Friedrich Alexander Universität, Erlangen Nürnberg, Medical Faculty, Department of Paediatric and Adolescents Medicine, Erlangen, Germany

^7^ CIO Marburg GmbH, Lohra, Germany

^8^ Department of Child and Adolescent Psychiatry, Medical University Vienna, Vienna, Austria

^9^ Department of Child and Adolescent Psychiatry, University of Ulm, Ulm, Germany

^10^ Department for Dermatology and Allergy, University Hospital RWTH Aachen, Aachen, Germany

**Corresponding author:**

Dr. Diana Dubrall

University of Bonn, University Hospital Bonn, Institute for Medical Biometry, Informatics and Epidemiology, Bonn, Germany

Research Division, Federal Institute of Drugs and Medical Devices (BfArM), Bonn, Germany

E-mail: Diana.Dubrall@bfarm.de

**Online Resource 3) Age-stratified analyses of ADHD drug prescriptions.**

**Online Resource 3 Table 1) Cumulative number of ADHD drug prescriptions** **between 2013 and 2022 stratified by age groups.**

| Rank | Pre-school children (< 6 years) | School children (6-12 years) | Adolescents (13-17 years) |
| --- | --- | --- | --- |
| 1.  2.  3.  4.  5. | Methylphenidate (n= 23,881)  Atomoxetine (n= 1,854)  Lisdexamfetamine (n= 1,339)  Guanfacine (n= 630)  Dexamfetamine (n= 451) | Methylphenidate (n= 5,974,006)  Lisdexamfetamine (n= 788,178)  Atomoxetine (n= 404,339)  Guanfacine (n= 134,083)  Dexamfetamine (n= 85,027) | Methylphenidate (n= 4,187,873)  Lisdexamfetamine (n= 750,757)  Atomoxetine (n= 309,154)  Guanfacine (n= 91,329)  Dexamfetamine (n= 66,323) |

Online Resource 3 Table 1 shows the total number of ADHD drug prescriptions between 2013 and 2022 for the three age groups of pre-school children, school children and adolescents. Notably, Guanfacine received approval in 2015, which may explain its lower counts. Data regarding guanfacine were available from 2020 onwards.

**Online Resource 3 Figure 1) Number of drug prescription stratified by ADHD drugs for pre-school children.**

Online Resource 3 Figure 1 shows the number of prescriptions of ADHD drugs per year for pre-school children. Notably, the number of methylphenidate prescriptions is shown on the secondary y-axis. Guanfacine received approval in 2015, which may explain its lower counts. Data regarding guanfacine were available from 2020 onwards.

**Online Resource 3 Figure 2) Number of drug prescription stratified by ADHD drugs for school children.**

Online Resource 3 Figure 2 shows the number of prescriptions of ADHD drugs per year for school children. Notably, the number of methylphenidate prescriptions is shown on the secondary y-axis. Guanfacine received approval in 2015, which may explain its lower counts. Data regarding guanfacine were available from 2020 onwards.

**Online Resource 3 Figure 3) Number of drug prescription stratified by ADHD drugs for adolescents.**

Online Resource 3 Figure 3 shows the number of prescriptions of ADHD drugs per year for adolescents. Notably, the number of methylphenidate prescriptions is shown on the secondary y-axis. Guanfacine received approval in 2015, which may explain its lower counts. Data regarding guanfacine were available from 2020 onwards.
