# Supplementary material for: Prescribing trends and spontaneous reporting patterns of adverse drug reactions of ADHD medications in children and adolescents in germany: A retrospective analysis (2013–2022)
Source: Eur Child Adolesc Psychiatry. 2026 Apr 27;35(8):2593–605. doi: 10.1007/s00787-026-03031-5 (PMC13652941; doi:10.1007/s00787-026-03031-5)
Supplement: Supplementary file 4 [file 787_2026_3031_MOESM4_ESM.docx]

**Title: Prescribing Trends and Spontaneous Reporting Patterns of Adverse Drug Reactions of ADHD Medications in Children and Adolescents in Germany: A Retrospective Analysis (2013-2022)**

**Journal: European Child & Adolescent Psychiatry**

**Authors:**

Diana Dubrall^1,2^, Mona Reifferscheid^3^, Karin Egberts^4,5^, Stefanie Fekete^4^, Antje Neubert^6^, Hans W Rock^7^, Paul Plener^8,9^, Maike Below^3^, Marcel Romanos^4^, Matthias Schmid^1^, Bernhardt Sachs^2,10^

^1^ University of Bonn, University Hospital Bonn, Institute for Medical Biometry, Informatics and Epidemiology, Bonn, Germany

^2^ Research Division, Federal Institute for Drugs and Medical Devices (BfArM), Bonn, Germany

^3^ Central Research Institute for Ambulatory Health Care in Germany, Berlin, Germany

^4^ Department of Child and Adolescent Psychiatry, Psychosomatics and Psychotherapy, Center of Mental Health, University Hospital of Wuerzburg, Wuerzburg, Germany

^5^ Department of Child and adolescent Psychiatry, GGZ Reinier van Arkel, ‘s-Hertogenbosch, The Netherlands

^6^ Friedrich Alexander Universität, Erlangen Nürnberg, Medical Faculty, Department of Paediatric and Adolescents Medicine, Erlangen, Germany

^7^ CIO Marburg GmbH, Lohra, Germany

^8^ Department of Child and Adolescent Psychiatry, Medical University Vienna, Vienna, Austria

^9^ Department of Child and Adolescent Psychiatry, University of Ulm, Ulm, Germany

^10^ Department for Dermatology and Allergy, University Hospital RWTH Aachen, Aachen, Germany

**Corresponding author:**

Dr. Diana Dubrall

University of Bonn, University Hospital Bonn, Institute for Medical Biometry, Informatics and Epidemiology, Bonn, Germany

Research Division, Federal Institute of Drugs and Medical Devices (BfArM), Bonn, Germany

E-mail: Diana.Dubrall@bfarm.de

**Online Resource 4) Sex-stratified analyses of ADHD drug prescriptions.**

**Online Resource 4 Table 1) Cumulative number of drug prescriptions for females and males between 2013 and 2022.**

| Rank | Females | Males |
| --- | --- | --- |
| 1.  2.  3.  4.  5. | Methylphenidate (n= 2,095,582)  Lisdexamfetamine (n= 265,104)  Atomoxetine (n= 151,143)  Guanfacine (n= 37,641)  Dexamfetamine (n= 27,782) | Methylphenidate (n= 8,090,178)  Lisdexamfetamine (n= 1,275,170)  Atomoxetine (n= 564,204)  Guanfacine (n= 188,401)  Dexamfetamine (n= 124,019) |

Online Resource 4 Table 1 shows the total number of ADHD drug prescriptions between 2013 and 2022 for females and males. Notably, Guanfacine received approval in 2015, which may explain its lower counts. Data regarding guanfacine were available from 2020 onwards.

**Online Resource 4 Figure 1) Number of ADHD prescriptions for males.**

Online Resource 4 Figure 1 shows the number of prescriptions of ADHD drugs per year for males. Notably, the number of methylphenidate prescriptions is shown on the secondary y-axis. Guanfacine received approval in 2015, which may explain its lower counts. Data regarding guanfacine were available from 2020 onwards.

**Online Resource 4 Figure 2) Number of ADHD prescriptions for females.**

Online Resource 4 Figure 2 shows the number of prescriptions of ADHD drugs per year for females. Notably, the number of methylphenidate prescriptions is shown on the secondary y-axis. Guanfacine received approval in 2015, which may explain its lower counts. Data regarding guanfacine were available from 2020 onwards.
