# Supplementary material for: Prescribing trends and spontaneous reporting patterns of adverse drug reactions of ADHD medications in children and adolescents in germany: A retrospective analysis (2013–2022)
Source: Eur Child Adolesc Psychiatry. 2026 Apr 27;35(8):2593–605. doi: 10.1007/s00787-026-03031-5 (PMC13652941; doi:10.1007/s00787-026-03031-5)
Supplement: Supplementary file 5 [file 787_2026_3031_MOESM5_ESM.docx]

**Title: Prescribing Trends and Spontaneous Reporting Patterns of Adverse Drug Reactions of ADHD Medications in Children and Adolescents in Germany: A Retrospective Analysis (2013-2022)**

**Journal: European Child & Adolescent Psychiatry**

**Authors:**

Diana Dubrall^1,2^, Mona Reifferscheid^3^, Karin Egberts^4,5^, Stefanie Fekete^4^, Antje Neubert^6^, Hans W Rock^7^, Paul Plener^8,9^, Maike Below^3^, Marcel Romanos^4^, Matthias Schmid^1^, Bernhardt Sachs^2,10^

^1^ University of Bonn, University Hospital Bonn, Institute for Medical Biometry, Informatics and Epidemiology, Bonn, Germany

^2^ Research Division, Federal Institute for Drugs and Medical Devices (BfArM), Bonn, Germany

^3^ Central Research Institute for Ambulatory Health Care in Germany, Berlin, Germany

^4^ Department of Child and Adolescent Psychiatry, Psychosomatics and Psychotherapy, Center of Mental Health, University Hospital of Wuerzburg, Wuerzburg, Germany

^5^ Department of Child and adolescent Psychiatry, GGZ Reinier van Arkel, ‘s-Hertogenbosch, The Netherlands

^6^ Friedrich Alexander Universität, Erlangen Nürnberg, Medical Faculty, Department of Paediatric and Adolescents Medicine, Erlangen, Germany

^7^ CIO Marburg GmbH, Lohra, Germany

^8^ Department of Child and Adolescent Psychiatry, Medical University Vienna, Vienna, Austria

^9^ Department of Child and Adolescent Psychiatry, University of Ulm, Ulm, Germany

^10^ Department for Dermatology and Allergy, University Hospital RWTH Aachen, Aachen, Germany

**Corresponding author:**

Dr. Diana Dubrall

University of Bonn, University Hospital Bonn, Institute for Medical Biometry, Informatics and Epidemiology, Bonn, Germany

Research Division, Federal Institute of Drugs and Medical Devices (BfArM), Bonn, Germany

E-mail: Diana.Dubrall@bfarm.de

**Online Resource 5) Descriptive analysis of spontaneous ADHD reports.**

**Online Resource 5 Figure 1) Number of ADR reports and ADHD reports per year.**

Online Resource 5 Figure 1 shows the number of spontaneous reports and the number of ADHD reports referring to children and adolescents per year. Notably, the number of spontaneous reports is shown on the secondary y-axis.

**Online Resource 5 Table 1) Descriptive analysis of ADHD reports received between 2013 and 2022.**

|  | ADHD reports (n= 1,119) |
| --- | --- |
| Age groups of the patients^1^ | |
| Pre-school children  School children  Adolescents | 0.4% (n= 5)  63.2% (n= 707)  36.4% (n= 407) |
| Sex of the patients | |
| Female  Males  Unknown | 20.3% (n= 227)  76.0% (n= 850)  3.8% (n= 42) |
| Seriousness^2^ | |
| Serious  Non-serious  Hospitalisation | 41.6% (n= 466)  58.4% (n= 653)  14.9% (n= 167) |
| Reporting source^3^ | |
| Physicians  Pharmacist  Other HCP  Consumer | 48.6% (n= 544)  7.5% (n= 84)  4.2% (n= 47)  17.4% (n= 195) |
| ADHD drugs reported as suspected/interacting^4^ | |
| 1.  2.  3.  4.  5. | Methylphenidate (60.1%, n= 673)  Lisdexamfetamine (21.4%, n= 240)  Atomoxetine (11.0%, n= 123)  Guanfacine (8.6%, n= 96)  Dexamfetamine (2.1%, n= 24) |

^1^ For the age groups, the patients were classified into pre-school children (< 6 years), school children (6-12 years), and adolescents (> 12 years).

^2^ more than one seriousness criterion can be reported in each ADR report. The evaluation of seriousness follows the legal definition. An ADR report is classified as serious, if the ADR was life-threatening, led to death, disabilities, congenital anomalies or hospitalisation or prolongation thereof.

^3^the primary source qualification describes the person who created the ADR report. One ADR report can be reported by more than one person with more than one qualification (e.g. physician and consumer reporting independently or together the same ADR). Shown is the number of reports explicitly reported by a one primary reporting source. In 249 reports more than one primary reporting source was recorded.

^4^ more than one drug could be reported as suspected/interacting.

Online Resource 5 Table 1 presents the descriptive analysis of the spontaneous ADHD reports. Thereby, the ADHD reports were investigated regarding the age and sex of the patients, the classification of seriousness, their reporting source, and their most frequently reported suspected/interacting ADHD drugs.

**Online Resource 5 Figure 2) Number of reports from different reporters per year.**

Online Resource 5 Figure 2 shows the number of spontaneous ADHD reports reported by physicians, pharmacists, consumers and non-HCP per year.

**Online Resource 5 Figure 3) Number of reports stratified by seriousness.**

Online Resource 5 Figure 3 shows the number of spontaneous ADHD reports classified as serious, non-serious and those coded with hospitalization (included in the number of serious reports).

**Online Resource 5 Figure 4) Number of serious reports stratified by ADHD drugs per year.**

Online Resource 5 Figure 4 shows the number of serious ADR reports for the individual ADHD drugs per year.

**Online Resource 5 Figure 5) Number of serious reports from different reporters per year**

Online Resource 5 Figure 5 shows the number of serious ADHD reports reported by physicians, pharmacists, consumers and non-HCP per year.

**Online Resource 5 Figure 6) Number of serious reports stratified by some of the most frequently reported ADRs.**

Online Resource 5 Figure 6 shows the number of serious ADHD reports stratified by some of the most frequently reported ADRs per year.

**Online Resource 5 Figure 7) Number of reports stratified by sex per year.**

Online Resource 5 Figure 7 shows the number of spontaneous ADHD reports referring to females, males and individuals for whom sex was reported as unknown.

**Online Resource 5 Figure 8) Number of reports stratified by age groups per year.**

Online Resource 5 Figure 8 shows the number of spontaneous ADHD reports referring to pre-school children, school children and adolescents. For the age groups, the patients were classified into pre-school children (< 6 years), school children (6-12 years), and adolescents (> 12 years).
