# Supplementary material for: Prescribing trends and spontaneous reporting patterns of adverse drug reactions of ADHD medications in children and adolescents in germany: A retrospective analysis (2013–2022)
Source: Eur Child Adolesc Psychiatry. 2026 Apr 27;35(8):2593–605. doi: 10.1007/s00787-026-03031-5 (PMC13652941; doi:10.1007/s00787-026-03031-5)
Supplement: Supplementary file 6 [file 787_2026_3031_MOESM6_ESM.docx]

**Title: Prescribing Trends and Spontaneous Reporting Patterns of Adverse Drug Reactions of ADHD Medications in Children and Adolescents in Germany: A Retrospective Analysis (2013-2022)**

**Journal: European Child & Adolescent Psychiatry**

**Authors:**

Diana Dubrall^1,2^, Mona Reifferscheid^3^, Karin Egberts^4,5^, Stefanie Fekete^4^, Antje Neubert^6^, Hans W Rock^7^, Paul Plener^8,9^, Maike Below^3^, Marcel Romanos^4^, Matthias Schmid^1^, Bernhardt Sachs^2,10^

^1^ University of Bonn, University Hospital Bonn, Institute for Medical Biometry, Informatics and Epidemiology, Bonn, Germany

^2^ Research Division, Federal Institute for Drugs and Medical Devices (BfArM), Bonn, Germany

^3^ Central Research Institute for Ambulatory Health Care in Germany, Berlin, Germany

^4^ Department of Child and Adolescent Psychiatry, Psychosomatics and Psychotherapy, Center of Mental Health, University Hospital of Wuerzburg, Wuerzburg, Germany

^5^ Department of Child and adolescent Psychiatry, GGZ Reinier van Arkel, ‘s-Hertogenbosch, The Netherlands

^6^ Friedrich Alexander Universität, Erlangen Nürnberg, Medical Faculty, Department of Paediatric and Adolescents Medicine, Erlangen, Germany

^7^ CIO Marburg GmbH, Lohra, Germany

^8^ Department of Child and Adolescent Psychiatry, Medical University Vienna, Vienna, Austria

^9^ Department of Child and Adolescent Psychiatry, University of Ulm, Ulm, Germany

^10^ Department for Dermatology and Allergy, University Hospital RWTH Aachen, Aachen, Germany

**Corresponding author:**

Dr. Diana Dubrall

University of Bonn, University Hospital Bonn, Institute for Medical Biometry, Informatics and Epidemiology, Bonn, Germany

Research Division, Federal Institute of Drugs and Medical Devices (BfArM), Bonn, Germany

E-mail: Diana.Dubrall@bfarm.de

**Online Resource 6) Stratified analysis of reporting rates**

**Online Resource 6 Table 1) Reporting rates per 100,000 prescriptions between 2013-2022.**

| Reporting rates | Methylphenidate | Lisdexamfetamine | Atomoxetine | Guanfacine^1^ | Dexamfetamine^2^ |
| --- | --- | --- | --- | --- | --- |
| Mean (±SD) | 6.7 (±3.1) | 22.2 (±19.9) | 17.8 (±8.2) | 19.7 (±7.2) | 17.3 (±13.0) |
| Median [IQR] | 5.6 [4.1-9.0] | 15.6 [12.4-26.0] | 17.5 [12.3-22.5] | 21.6 [17.4-23.8] | 17.3 [7.4-26.7] |

SD= standard deviation, IQR= interquartile range

^1^drug prescription data for guanfacine were available from 2020 onwards. Thus, reporting rates could only be calculated for the years 2020, 2021 and 2022.

^2^the first ADHD report concerning dexamfetamine was received in 2015. Thus, reporting rates could only be calculated between 2015 and 2022.

Online Resource 6 Table 1 shows the mean and median number and their SD and IQR of ADHD reports per 100,000 drug prescriptions between 2013 and 2022.

Online Resource 6 Figure 1) Reporting rates per year stratified by individual ADHD drugs.

Online Resource 6 Figure 1 shows the reporting rates for the individual ADHD drugs per year. For calculation of reporting rates, the number of spontaneous ADHD reports was divided by the number of outpatient ADHD prescriptions. The reporting rates are given as the number of reports per 100,000 drug prescriptions.

**Online Resource 6 Table 2) Reporting rates of serious ADRs per 100,000 prescriptions between 2013-2022.**

| Reporting rates | Methylphenidate | Lisdexamfetamine | Atomoxetine | Guanfacine^1^ | Dexamfetamine^2^ |
| --- | --- | --- | --- | --- | --- |
| Mean (±SD) | 2.6 (±0.9) | 11.5 (±13.4) | 7.0 (±4.8) | 7.6 (±3.6) | 3.1 (±8.4) |
| Median [IQR] | 2.4 [2.2-3.1] | 5.5 [1.9-18.1] | 5.4 [3.7-9.9] | 8.1 [5.9-9.5] | 0.0 [0.0-0.0] |

SD= standard deviation, IQR= interquartile range

^1^drug prescription data for guanfacine were available from 2020 onwards. Thus, reporting rates could only be calculated for the years 2020, 2021 and 2022.

^2^serious ADR reports concerning dexamfetamine were only received in 2015 and 2020.

Online Resource 6 Table 2 shows the mean and median number and their SD and IQR of serious ADHD reports per 100,000 drug prescriptions between 2013 and 2022.

Online Resource 6 Figure 2) Reporting rates of serious ADRs per year stratified by individual ADHD drugs.

Online Resource 6 Figure 2 shows the reporting rates of serious ADRs for the individual ADHD drugs per year. For calculation of reporting rates, the number of spontaneous ADHD reports was divided by the number of outpatient ADHD prescriptions. The reporting rates are given as the number of reports per 100,000 drug prescriptions.

**Online Resource 6 Table 3) Reporting rates per 100,000 prescriptions between 2013-2022 stratified by age groups.**

| Reporting rates | Methylphenidate | Lisdexamfetamine | Atomoxetine | Guanfacine# |
| --- | --- | --- | --- | --- |
| Mean (±SD)  School children  Adolescents | 6.7 (±3.1)  7.2 (±3.4)  5.9 (±2.9) | 22.2 (±19.9)  24.2 (±19.9)  18.4 (±16.1) | 17.8 (±8.2)  19.6 (±11.7)  15.3 (±5.3) | 19.7 (±7.2)  22.5 (±8.2)  15.9 (±14.3) |
| Median [IQR]  School children  Adolescents | 5.6 [4.1-9.0]  6.6 [4.0-10.2]  5.6 [3.8-7.5} | 15.6 [12.4-26.0]  18.0 [15.5-27.0]  18.3 [7.9-21.7] | 17.5 [12.3-22.5]  18.3 [10.1-25.0]  15.4 [13.0-17.9] | 21.6 [17.4-23.8]  18.3 [17.7-25.2]  13.2 [8.1-22.2] |

^1^drug prescription data for guanfacine were available from 2020 onwards. Thus, reporting rates could only be calculated for the years 2020, 2021 and 2022.

Online Resource 6 Table 3 shows the mean and median number and their SD and IQR of ADHD reports per 100,000 drug prescriptions between 2013 and 2022 for school children and adolescents. The number of reports referring to pre-school children was too small (n= 5) to allow further stratification.

**Online Resource 6 Figure 3) Reporting rates of methylphenidate per 100,000 drug prescriptions stratified by age groups.**

Online Resource 6 Figure 3 shows the reporting rates of methylphenidate per year for school children and adolescents. For calculation of reporting rates, the number of spontaneous ADHD reports was divided by the number of outpatient ADHD prescriptions. For temporal analysis of the reporting rates Poisson or negative binomial models with cubic splines were used to account for non-linear changes over time with drug prescription counts as offset to adjust for drug exposure. The reporting rates are given as the number of reports per 100,000 drug prescriptions.

**Online Resource 6 Figure 4 Reporting rates of atomoxetine per 100,000 drug prescriptions stratified by age groups.**

Online Resource 6 Figure 4 shows the reporting rates of atomoxetine per year for school children and adolescents. For calculation of reporting rates, the number of spontaneous ADHD reports was divided by the number of outpatient ADHD prescriptions. For temporal analysis of the reporting rates Poisson or negative binomial models with cubic splines were used to account for non-linear changes over time with drug prescription counts as offset to adjust for drug exposure. The reporting rates are given as the number of reports per 100,000 drug prescriptions.

**Online Resource 6 Figure 5) Reporting rates of lisdexamfetamine per 100,000 drug prescriptions stratified by age groups.**

Online Resource 6 Figure 5 shows the reporting rates of lisdexamfetamine per year for school children and adolescents. For calculation of reporting rates, the number of spontaneous ADHD reports was divided by the number of outpatient ADHD prescriptions. For temporal analysis of the reporting rates Poisson or negative binomial models with cubic splines were used to account for non-linear changes over time with drug prescription counts as offset to adjust for drug exposure. The reporting rates are given as the number of reports per 100,000 drug prescriptions.

**Online Resource 6 Figure 6) Reporting rates of guanfacine per 100,000 drug prescriptions stratified by age groups.**

Online Resource 6 figure 6) shows the reporting rates of guanfacine per year for school children and adolescents. For calculation of reporting rates, the number of spontaneous ADHD reports was divided by the number of outpatient ADHD prescriptions. The reporting rates are given as the number of reports per 100,000 drug prescriptions. Guanfacine received approval in 2015, however, drug prescription data were available from 2020 onwards. Thus, reporting rates could only be calculated for 2020, 2021 and 2022.

Online Resource 6 Table 4) Reporting rates per 100,000 prescriptions between 2013-2022 stratified by sex.

| Reporting rates | Methylphenidate | Lisdexamfetamine | Atomoxetine | Guanfacine^1^ |
| --- | --- | --- | --- | --- |
| Mean (±SD)  Females  Males | 6.7 (±3.1)  7.1 (±3.2)  6.3 (±2.8) | 22.2 (±19.9)  18.2 (±9.7)  21.7 (±21.0) | 17.8 (±8.2)  16.6 (±12.8)  17.4 (±9.0) | 19.7 (±7.2)  24.0 (±1.6)  18.2 (±8.0) |
| Median [IQR]  Females  Males | 5.6 [4.1-9.0]  6.1 [5.3-8.2]  5.6 [3.9-9.0} | 15.6 [12.4-26.0]  16.8 [9.8-26.7]  14.1 [12.0-25.3] | 17.5 [12.3-22.5]  15.1 [6.3-22.0]  17.3 [11.8-21.7] | 21.6 [17.4-23.8]  24.0 [23.2-24.8]  21.4 [15.3-22.8] |

^1^drug prescription data for guanfacine were available from 2020 onwards. Thus, reporting rates could only be calculated for the years 2020, 2021 and 2022.

Online Resource 6 Table 4 shows the mean and median number and their SD and IQR of ADHD reports per 100,000 drug prescriptions between 2013 and 2022 for females and males.

**Online Resource 6 Figure 7) Reporting rates of methylphenidate per 100,000 drug prescriptions stratified by sex.**

Online Resource 6 Figure 7 shows the reporting rates of methylphenidate per year for females and males. For calculation of reporting rates, the number of spontaneous ADHD reports was divided by the number of outpatient ADHD prescriptions. For temporal analysis of the reporting rates Poisson or negative binomial models with cubic splines were used to account for non-linear changes over time with drug prescription counts as offset to adjust for drug exposure. The reporting rates are given as the number of reports per 100,000 drug prescriptions.

**Online Resource 6 Figure 8) Reporting rates of atomoxetine per 100,000 drug prescriptions stratified by sex.**

Online Resource 6 Figure 8 shows the reporting rates of atomoxetine per year for females and males. For calculation of reporting rates, the number of spontaneous ADHD reports was divided by the number of outpatient ADHD prescriptions. For temporal analysis of the reporting rates Poisson or negative binomial models with cubic splines were used to account for non-linear changes over time with drug prescription counts as offset to adjust for drug exposure. The reporting rates are given as the number of reports per 100,000 drug prescriptions.

**Online Resource 6 Figure 9) Reporting rates of lisdexamfetamine per 100,000 drug prescriptions stratified by sex.**

Online Resource 6 Figure 9 shows the reporting rates of lisdexamfetamine per year for females and males. For calculation of reporting rates, the number of spontaneous ADHD reports was divided by the number of outpatient ADHD prescriptions. For temporal analysis of the reporting rates Poisson or negative binomial models with cubic splines were used to account for non-linear changes over time with drug prescription counts as offset to adjust for drug exposure. The reporting rates are given as the number of reports per 100,000 drug prescriptions.

**Online Resource 6 Figure 10) Reporting rates of guanfacine per 100,000 drug prescriptions stratified by sex.**

Online Resource 6 Figure 10 shows the reporting rates of guanfacine per year for females and males. For calculation of reporting rates, the number of spontaneous ADHD reports was divided by the number of outpatient ADHD prescriptions. The reporting rates are given as the number of reports per 100,000 drug prescriptions. Guanfacine received approval in 2015, however, drug prescription data were available from 2020 onwards. Thus, reporting rates could only be calculated for 2020, 2021 and 2022.
