# Supplementary material for: Prescribing trends and spontaneous reporting patterns of adverse drug reactions of ADHD medications in children and adolescents in germany: A retrospective analysis (2013–2022)
Source: Eur Child Adolesc Psychiatry. 2026 Apr 27;35(8):2593–605. doi: 10.1007/s00787-026-03031-5 (PMC13652941; doi:10.1007/s00787-026-03031-5)
Supplement: Supplementary file 7 [file 787_2026_3031_MOESM7_ESM.docx]

**Title: Prescribing Trends and Spontaneous Reporting Patterns of Adverse Drug Reactions of ADHD Medications in Children and Adolescents in Germany: A Retrospective Analysis (2013-2022)**

**Journal: European Child & Adolescent Psychiatry**

**Authors:**

Diana Dubrall^1,2^, Mona Reifferscheid^3^, Karin Egberts^4,5^, Stefanie Fekete^4^, Antje Neubert^6^, Hans W Rock^7^, Paul Plener^8,9^, Maike Below^3^, Marcel Romanos^4^, Matthias Schmid^1^, Bernhardt Sachs^2,10^

^1^ University of Bonn, University Hospital Bonn, Institute for Medical Biometry, Informatics and Epidemiology, Bonn, Germany

^2^ Research Division, Federal Institute for Drugs and Medical Devices (BfArM), Bonn, Germany

^3^ Central Research Institute for Ambulatory Health Care in Germany, Berlin, Germany

^4^ Department of Child and Adolescent Psychiatry, Psychosomatics and Psychotherapy, Center of Mental Health, University Hospital of Wuerzburg, Wuerzburg, Germany

^5^ Department of Child and adolescent Psychiatry, GGZ Reinier van Arkel, ‘s-Hertogenbosch, The Netherlands

^6^ Friedrich Alexander Universität, Erlangen Nürnberg, Medical Faculty, Department of Paediatric and Adolescents Medicine, Erlangen, Germany

^7^ CIO Marburg GmbH, Lohra, Germany

^8^ Department of Child and Adolescent Psychiatry, Medical University Vienna, Vienna, Austria

^9^ Department of Child and Adolescent Psychiatry, University of Ulm, Ulm, Germany

^10^ Department for Dermatology and Allergy, University Hospital RWTH Aachen, Aachen, Germany

**Corresponding author:**

Dr. Diana Dubrall

University of Bonn, University Hospital Bonn, Institute for Medical Biometry, Informatics and Epidemiology, Bonn, Germany

Research Division, Federal Institute of Drugs and Medical Devices (BfArM), Bonn, Germany

E-mail: Diana.Dubrall@bfarm.de

**Online Resource 7) Reporting rates in relation to the number of patients receiving drug prescriptions.**

**Online Resource 7 Table 1) Reporting rates per 100,000 patients with ADHD drugs prescriptions between 2013 and 2022.**

| Reporting rates | Methylphenidate | Lisdexamfetamine | Atomoxetine | Guanfacine^1^ | Dexamfetamine^2^ |
| --- | --- | --- | --- | --- | --- |
| Mean (±SD) | 37.8 (±17.1) | 101.6 (±53.1) | 105.2 (±48.4) | 135.3 (±50.0) | 76.3 (±58.5) |
| Median [IQR] | 31.9 [23.2-50.3] | 87.6 [71.7-135.5] | 100.9 [74.0-134.1] | 161.6 [119.6-164.1] | 72.4 33.2-110.3] |

SD= standard deviation, IQR= interquartile range

^1^the numbers of patients with guanfacine prescriptions were available from 2020 onwards. Thus, reporting rates could only be calculated for the years 2020, 2021, and 2022.

^2^the first ADHD report concerning dexamfetamine was received in 2015. Thus, reporting rates could only be calculated between 2015 and 2022.

Online Resource 7 Table 1 shows the mean and median number and their SD and IQR of ADHD reports per 100,000 patients with drug prescriptions between 2013 and 2022.

Online Resource 7 Figure 1) Reporting rates per 100,000 patients with ADHD drug prescriptions stratified by individual ADHD drugs.

Online Resource 7 Figure 1 shows the reporting rates in relation to the number of patients with ADHD drug prescriptions for the individual ADHD drugs per year. For calculation of reporting rates, the number of spontaneous ADHD reports was divided by the number of patients with outpatient ADHD prescriptions. The reporting rates are given as the number of reports per 100,000 patients with drug prescriptions.

**Online Resource 7 Table 2) Reporting rates per 100,000 prescriptions between 2013-2022 stratified by age groups.**

| Reporting rates | Methylphenidate | Lisdexamfetamine | Atomoxetine | Guanfacine# |
| --- | --- | --- | --- | --- |
| Mean (±SD)  School children  Adolescents | 37.8 (±17.1)  42.6 (±19.7)  31.4 (±15.3) | 101.6 (±53.1)  113.8 (±54.7)  83.1 (±46.3) | 105.2 (±48.4)  119.8 (±70.5)  87.2 (±29.7) | 135.3 (±50.0)  150.7 (±55.5)  114.0 (±103.1) |
| Median [IQR]  School children  Adolescents | 31.9 [23.2-50.3]  39.0 [23.8-59.6]  29.8 [20.7-39.5} | 87.6 [71.7-135.5]  105.9 [89.3-150.8]  73.8 [44.5-106.7] | 100.9 [74.0-134.1]  110.4 [62.1-156.5]  88.0 [73.7-103.1] | 161.6 [119.6-164.1]  123.3 [118.7-168.9]  94.5 [58.3-160.0] |

^1^the numbers of patients with guanfacine prescriptions were available from 2020 onwards. Thus, reporting rates could only be calculated for the years 2020, 2021, and 2022.

Online Resource 7 Table 2 shows the mean and median number and their SD and IQR of ADHD reports per 100,000 patients with outpatient ADHD prescriptions between 2013 and 2022 for school children and adolescents. The number of reports referring to pre-school children was too small (n= 5) to allow further stratification.

**Online Resource 7 Figure 2) Reporting rates of methylphenidate per 100,000 patients with methylphenidate prescriptions stratified by age groups.**

Online Resource 7 Figure 2) shows the reporting rates related to the number of school children and adolescents with methylphenidate prescriptions per year. For calculation of reporting rates, the number of spontaneous ADHD reports was divided by the number of patients with outpatient methylphenidate prescriptions. The reporting rates are given as the number of reports per 100,000 patients with methylphenidate prescriptions.

**Online Resource 7 Figure 3) Reporting rates of atomoxetine per 100,000 patients with atomoxetine prescriptions stratified by age groups.**

Online Resource 7 Figure 3 shows the reporting rates related to the number of school children and adolescents with atomoxetine prescriptions per year. For calculation of reporting rates, the number of spontaneous ADHD reports was divided by the number of patients with outpatient atomoxetine prescriptions. The reporting rates are given as the number of reports per 100,000 patients with atomoxetine prescriptions.

**Online Resource 7 Figure 4) Reporting rates of lisdexamfetamine per 100,000 patients with lisdexamfetamine prescriptions stratified by age groups.**

Online Resource 7 Figure 4 shows the reporting rates related to the number of school children and adolescents with lisdexamfetamine prescriptions per year. For calculation of reporting rates, the number of spontaneous ADHD reports was divided by the number of patients with outpatient lisdexamfetamine prescriptions. The reporting rates are given as the number of reports per 100,000 patients with lisdexamfetamine prescriptions.

**Online Resource 7 Figure 5) Reporting rates of guanfacine per 100,000 patients with guanfacine prescriptions stratified by age groups.**

Online Resource 7 Figure 5 shows the reporting rates related to the number of school children and adolescents with guanfacine prescriptions per year. For calculation of reporting rates, the number of spontaneous ADHD reports was divided by the number of patients with outpatient guanfacine prescriptions. The reporting rates are given as the number of reports per 100,000 patients with guanfacine prescriptions. Guanfacine received approval in 2015, however, drug prescription data were available from 2020 onwards. Thus, reporting rates could only be calculated for 2020, 2021 and 2022.

**Online Resource 7 Table 3) Reporting rates per 100,000 patients with ADHD drugs prescriptions between 2013 and 2022 stratified by sex.**

| Reporting rates | Methylphenidate | Lisdexamfetamine | Atomoxetine | Guanfacine^1^ |
| --- | --- | --- | --- | --- |
| Mean (±SD)  Females  Males | 37.8 (±17.1)  37.7 (±16.7)  35.9 (±15.6) | 101.6 (±53.1)  86.5 (±42.4)  99.1 (±58.0) | 105.2 (±48.4)  93.8 (±71.2)  104.3 (±54.4) | 135.3 (±50.0)  157.3 (±10.4)  126.7 (±77.7) |
| Median [IQR]  Females  Males | 31.9 [23.2-50.3]  33.0 [28.4-42.8]  31.9 [22.9-51.6] | 87.6 [71.7-135.5]  86.5 [52.1-101.4]  81.5 [69.6-138.7] | 100.9 [74.0-134.1]  87.5 [36.7-122.5]  101.2 [71.2-134.3] | 161.6 [119.6-164.1]  159.2 [152.6-163.0]  149.1 [106.0-158.6] |

^1^the numbers of patients with guanfacine prescriptions were available from 2020 onwards. Thus, reporting rates could only be calculated for the years 2020, 2021, and 2022.

Online Resource 7 Table 3 shows the mean and median number and their SD and IQR of ADHD reports per 100,000 patients with ADHD drug prescriptions between 2013 and 2022 for females and males.

**Online Resource 7 Figure 6) Reporting rates of methylphenidate per 100,000 patients with methylphenidate prescriptions stratified by sex.**

Online Resource 7 Figure 6 shows the reporting rates related to the number of females and males with methylphenidate prescriptions per year. For calculation of reporting rates, the number of spontaneous ADHD reports was divided by the number of patients with outpatient methylphenidate prescriptions. The reporting rates are given as the number of reports per 100,000 patients with methylphenidate prescriptions.

**Online Resource 7 Figure 7) Reporting rates of atomoxetine per 100,000 patients with atomoxetine prescriptions stratified by sex.**

Online Resource 7 Figure 7 shows the reporting rates related to the number of females and males with atomoxetine prescriptions per year. For calculation of reporting rates, the number of spontaneous ADHD reports was divided by the number of patients with outpatient atomoxetine prescriptions. The reporting rates are given as the number of reports per 100,000 patients with atomoxetine prescriptions.

**Online Resource 7 Figure 8) Reporting rates of lisdexamfetamine per 100,000 patients with lisdexamfetamine prescriptions stratified by sex.**

Online Resource 7 Figure 8 shows the reporting rates related to the number of females and males with lisdexamfetamine prescriptions per year. For calculation of reporting rates, the number of spontaneous ADHD reports was divided by the number of patients with outpatient lisdexamfetamine prescriptions. The reporting rates are given as the number of reports per 100,000 patients with lisdexamfetamine prescriptions.

**Online Resource 7 Figure 9) Reporting rates of guanfacine per 100,000 patients with guanfacine prescriptions stratified by sex.**

Online Resource 7 Figure 9 shows the reporting rates related to the number of females and males with guanfacine prescriptions per year. For calculation of reporting rates, the number of spontaneous ADHD reports was divided by the number of patients with outpatient guanfacine prescriptions. The reporting rates are given as the number of reports per 100,000 patients with guanfacine prescriptions. Guanfacine received approval in 2015, however, drug prescription data were available from 2020 onwards. Thus, reporting rates could only be calculated for 2020, 2021 and 2022.
